# Non-Local Parallel Processing and Database Settlement Using Multiple Teleportation Followed by Grover Post-Selection

**DOI:** 10.3390/e25020376

**Published:** 2023-02-18

**Authors:** Francisco Delgado, Carlos Cardoso-Isidoro

**Affiliations:** 1 Tecnologico de Monterrey, School of Engineering and Science, Atizapan 52926, Mexico; 2 Tecnologico de Monterrey, School of Engineering and Science, Monterrey 64849, Mexico

**Keywords:** quantum databases, parallel processing, quantum multiple teleportation, Grover algorithm, non-local control, quantum intelligence

## Abstract

Quantum information applications emerged decades ago, initially introducing a parallel development that mimicked the approach and development of classical computer science. However, in the current decade, novel computer-science concepts were rapidly extended to the fields of quantum processing, computation, and communication. Thus, areas such as artificial intelligence, machine learning, and neural networks have their quantum versions; furthermore, the quantum brain properties of learning, analyzing, and gaining knowledge are discussed. Quantum properties of matter conglomerates have been superficially explored in such terrain; however, the settlement of organized quantum systems able to perform processing can open a new pathway in the aforementioned domains. In fact, quantum processing involves certain requisites as the settlement of copies of input information to perform differentiated processing developed far away or in situ to diversify the information stored there. Both tasks at the end provide a database of outcomes with which to perform either information matching or final global processing with at least a subset of those outcomes. When the number of processing operations and input information copies is large, parallel processing (a natural feature in quantum computation due to the superposition) becomes the most convenient approach to accelerate the database settlement of outcomes, thus affording a time advantage. In the current study, we explored certain quantum features to realize a speed-up model for the entire task of processing based on a common information input to be processed, diversified, and finally summarized to gain knowledge, either in pattern matching or global information availability. By using superposition and non-local properties, the most valuable features of quantum systems, we realized parallel local processing to set a large database of outcomes and subsequently used post-selection to perform an ending global processing or a matching of information incoming from outside. We finally analyzed the details of the entire procedure, including its affordability and performance. The quantum circuit implementation, along with tentative applications, were also discussed. Such a model could be operated between large processing technological systems using communication procedures and also on a moderately controlled quantum matter conglomerate. Certain interesting technical aspects involving the non-local control of processing via entanglement were also analyzed in detail as an associated but notable premise.

## 1. Introduction

Despite light being used to prove many features in quantum information and quantum processing, the use of matter will open a vast terrain for growing quantum applications. The current quantum processors use a limited number of systems as qubits; however, a small amount of matter, when controlled, can dramatically increase the quantum storage of information. There, quantum elements are near arranged; thus, non-local features can provide novel ways of processing, which is currently being performed by quantum communication.

An increasing number of knowledge areas and disciplines consider interactions with quantum information, communication, and processing to solve complex simulation problems, to obtain improved security or reproduce processes of machine learning or artificial intelligence. Recently, the term quantum cognition (QC) was coined to focus on the brain function quantum phenomena through disruptive research [1,2]. Such an area deals with the processes of perception, apprehension, comprehension, cognition, and decision making, particularly regarding quantum mechanics associated with the human brain [3]. Quantum logic is settled by the properties of quantum operators mainly representing observables, which become conjugate variables and subsequently Pontryagin duals [4], as is clearly illustrated by the Dirac three polarizers experiment [5]. Furthermore, the quantum Zeno’s effect [6,7] exhibiting superposition states and quantum entanglement for composed systems are considered, both of which feature quantum mechanics.

Traditional computing approaches are centered on sequential processing based on the Turing machine, still based on parallel processing (used as a procedure for acceleration). For instance, machine learning procedures still follow a linear procedure of composed steps that considers layers that, in the best case, probably considers parallelism or recursion [8,9]. Such approaches have reached quantum computation despite the fact that they only mildly exploit superposition and entanglement. While cognitive brain function is traditionally believed to be based on Bayesian inference through the free energy minimization principle, it still appears in conflict with QC owing to removal of the redundant search space, thereby moving toward non-optimal decision making. In fact, when the brain learns certain information, it can still infer contextual information to reach a desired outcome under a different situation, which considers different information from that initially and concretely owned. This implies, in parallel processing, a large amount of alternatives is selected until a final decision is made [2].

Nevertheless, new quantum computation models [10,11,12] have emerged after the circuit model based on universal gates, which is widely used. Following the trend, recently, a new paradigm of quantum computing-denominated quantum intelligence (QC) [13], which considers QC as a wider usage of quantum features in the brain function, has been proposed. In this approach, Bayesian inference is not applied in excess, leading to the inclusion of complementary events with non-zero probability, as in the classical version where events commonly outside the problem context are considered with zero probability. Such a difference allows for a wider context in the process of cognition that is closer to the real observed brain function closer to fuzzy systems [13,14].

The last fact suggests that wide-spreading knowledge boosts the simultaneous generation of processed alternatives to be selected later. With regard to quantum theory, this suggests that a large superposition of outcomes still maintain correlated links upon their selection. Thus, the settlement of a large database of processed outcomes departing from a single limited information diversified through processing operations (along with integration of concrete local information) should be considered as an elementary quantum process for reaching a global outcome or matching certain external information.

In quantum processing, such a database implies establishing differentiated information suitably codified on a set of quantum states. When the last operations are barely independent, parallel processing should be conveniently introduced owing to the number of tasks. Parallel processing has been pursued as a solution to certain complex problems based on our current computer technology. Such processing can be understood in the context of either classical or quantum systems. In classical systems, we can reduce the time required to solve a task by using certain information processing units simultaneously, whereas in the quantum realm, all of the input information can be addressed and combined into a single register, where such a quantum unit can still process all of them in only one step [15]. Thus, quantum computing has been hailed as an efficient massively parallel computing scheme [16].

The parallelism in quantum computing essentially relies on the ability of a physical system to exist in a superposition of states, thus allowing for a massively parallel solution of specific problems that are not efficiently handled by a classical computer, such as integer factorization performed based on quantum theory using the Shor algorithm [17]. Otherwise, the settlement of large quantum databases is particularly useful in quantum image processing (QIMP) and, in general, in quantum pattern matching (QPM) [18,19]. Considered as the foundation of quantum computation and quantum communication, teleportation is a process of quantum information transfer in the form of a quantum state from a system into another [20,21] and not limited to larger system states [22]. Teleportation becomes a useful quantum tool to send information on faraway receivers; moreover, alternatives for close systems in matter conglomerates are available. In addition, controlling such algorithm is feasible to selectively send information to several parties under superposition [23]. Such information transference can be exploited to set a large and diversified number of parallel processing operations using the same input. The outcomes can be stored in a temporary database through superposition.

Classical decision making regards the selection of information through Bayesian inference; however, quantum mechanics still provides privileged access to parts of classified information under dynamical processes where all such parts can play a role until a tentative measurement is acquired. Thus, in terms of quantum processing, such a selection of information can be performed through measurement or amplitude amplification, as exemplified by the Grover algorithm [24]. Such selection is useful for either performing a final global processing, extracting global information, or matching external information. Suitable and specific algorithms to codify, combine, and extract such global information incoming from the database outcomes can be implemented using only few steps owing to the state superposition and entanglement remaining in the global system. Such processes appear nearer to those considered by QC and QI.

The aim of the current study is to explore and analyze a multi-step quantum model to establish a database of outcomes obtained by chaining teleportation and subsequently parallel processing. The dataset settlement and further parallel processing on a local memory are classically and commonly waived. Based on alternative quantum approaches for both problems, we present a non-local approach exploiting the main features of Quantum Mechanics. Thus, the proposal is strongly based on non-local features provided by quantum systems. Such a process improves the classical issues present in classical database settlement in terms of communication, as discussed in the next section. This processing departs from a codified single qubit state as input, transferred in superposition on several systems working as processors via multiple quantum teleportation. The set of superposed outcomes fits the concept of a quantum database. Subsequently, a subset of them can be accessed through amplitude amplification. Finally, such a subset can be used for acquiring the stored global information or for information matching or query tasks. The process fits the feasible quantum artificial sensing and decision-making tasks, and it is also applied to explore a possible cellular process in QC and QI. The second section deals with the technical statement of the problem regarding classical concepts and issues around databases, parallel processing, and pattern matching. After discussing their possible advantages, the quantum versions are elaborated. This section also presents the global plot being presented along with the variables considered in the development. The third section presents the details of multiple teleportation and post-processing, thus setting the distributed database of outcomes. Furthermore, we discuss certain non-local procedures via entanglement to move the entire set of processing outcomes into one single party and continue with other possible ending procedures. Such important aspects consider teleportation processes for short or large distances (matter conglomerates or artificial processing systems). The third section discusses the post-selection process comprising stochastic post-selection and the subsequent analysis regarding the adaptation of Grover’s amplitude amplification algorithm to the last database outcomes in terms of recursive expressions, error quantification, and convergence under certain post-selection scenarios. The fourth section discusses several aspects related to the associated and translated errors from the previous amplitude amplification procedure on tentative further ending global processing or pattern matching. Certain aspects related to quantum circuit implementation are discussed, along with possible applications. The last section discusses the conclusions and future work.

## 2. Classical and Quantum Approaches for Parallel Processing on a Database Settlement

In this section, we set the classical concepts of database and parallel computing, discussing their possible issues in terms of future development, particularly those due to scalability in terms of the complexity of computing problems. Thereafter, quantum versions are discussed to introduce the current proposal.

### 2.1. The Classical Concepts of Database and Parallel Processing: Issues and Challenges

In classical computing, a database is a stored collection of data suitably organized to be electronically accessed, where organization for data access is realized through a register or index for each ordered set of data [25]. While databases have continuously grown, they are currently stored in computer clusters instead of simple data files; nonetheless, they are managed by software, a specialized database management system (DBMS). Data can be structured or otherwise ordered as a string; however, in any case, an explicit or implicit index is required. Databases could be stored *on-disk*, i.e., they may be enduring or permanent on a physical device. Otherwise, databases *in-memory* are a type of temporal set of data residing in the main memory of the processing device and can be accessed faster than the ones stored on disks.

Encoded in bits, a large number of data are settled temporarily on the main memory. Specialized software and algorithms attempt to optimize the access, processing, and queries on large databases. However, there are limits mainly pertaining to the physical media where data are stored: different places on the same device require specific logistics. Thus, their scalability with an increase in the database volume and their speedy access to information when multiple sets of data should be searched and used, naturally become contemporary challenges for classical databases in applications related to Data Science, particularly in genomics [26], which has stated new size limits.

In another trend, parallel processing or parallel computing is an approach in classical computation, where many of the involved computations in computer processes are performed simultaneously because they are commonly independent. Accordingly, the outcomes can be combined afterwards [27]. Despite the first approach of computation being serial computation, parallel processing is in fact a natural function in the brain. In fact, currently, the brain is considered a massively parallel computer [28]; thus in Psychology, the term parallel processing refers to the ability of the brain to simultaneously process several stimuli obtained through the senses [29]. Thus, parallel processing is in fact believed to be a natural way of processing in the brain. For this reason, the model presented could also mimic the brain behavior, upon elucidation of certain possible quantum features. In fact, because teleportation is a key element in the procedure to set a distributed database, it can be induced over short distances, not for telecommunication processes, rather for communication among chemical or still biological subsystems. In fact, as a consequence of entanglement, such effects are widely known in certain biological processes, such as bacteria, sensing optimized paths for energy transfer [30].

With the design of computers integrating more than one processor (the basic unit using incoming information to perform transformation on it by applying a defined computing procedure and possibly integrating additional information), parallel computing has become a more common approach to classical computation models involving 3D-movement in real time or simply any complex computation problem requiring improved polynomial runtimes at par with its scalability. Nevertheless, the speed-up introduced by parallelization is expected to be linear, and not all problems are completely parallelizable, bounding such linear behavior. In fact, it limits the performance in agreement with Amdahl’s law [31] stating the potential speed-up of an algorithm upon parallelization. The last limit is mainly associated with the impossibility to completely parallelize any problem; however, it also considers other factors referred to as access of information imposed on parallel processing.

### 2.2. Statement of the Current Database Settlement Problem in the Classical Approach

In a classical parallel computer, the main memory used to perform calculations is either a shared memory (among the processing elements located on a single storage space) or a distributed memory (where each processing data cluster has its own storage space). As expected, access to local memories is commonly faster than access to non-local memories [32]. Because all types of accesses are present in a typical parallelizable problem, physical or logical distribution imposes a natural slow down on scalability, doing speed communication the bottleneck of the process [32], either in the information access or in the necessary communication among computer parts during parallel processing.

Thus, in this work, we analyze a multi-step approach for parallel processing, which naturally demands the settlement of a database in a temporary memory. In the classical approach, the settlement, either regarding communication from permanent sources or information or simply the copying process of the main memory, to initialize parallel computing requires a sequential task based on use of a unique integrated control system. In the quantum approach, superposition improves such limitation. Copying, still limited by the no-cloning theorem, can be solved by multiple teleportation as a notable aspect, generating processable copies of a state in superposition to acquire from the outcomes an output with global information obtained by departing from the outcomes, before any measurement performed on the system.

### 2.3. Quantum Parallelism: Quantum Alternatives for Storage and Processing Systems

For scalability, storage and data access require an increasing additional runtime consumption owing to the limitations imposed by physical systems used in classical computing. Thus, database and parallel processing become related concepts integrating processing units which read data locally or non-locally to be involved repeatedly on specific computing tasks.

Alternative to classical databases, quantum databases refers to the use of quantum systems to store information. It is believed to solve limitations imposed by classical approaches due to superposition of physical states used as information containers. Superposition addresses both volume and access time [33]. In the quantum model being presented, we are considering an in-memory database, being temporal for a concrete and an ephemeral purpose involving processing, query, and post-processing.

For parallel processing, an alternative approach, quantum parallelism enables performing extensive calculations in parallel via superposition, thus stating a natural key advantage over classical computing in terms of the time and storage space. In addition, quantum systems still provide additional features boosting the possibility to introduce controlled non-local operations performed remotely through the quantum feature of entanglement [34]. In the current unit processing being analyzed, we exploit both quantum features, superposition, and entanglement, integrating the settlement of a distributed database combined with further differentiated processing to finally perform a query or acquire global information. That the model reproduces or suggests possible cognition tasks present in the brain proposed by disruptive models [13] suggests that the brain is a system possibly exhibiting quantum features in its macroscopic functioning.

**Figure 1:**
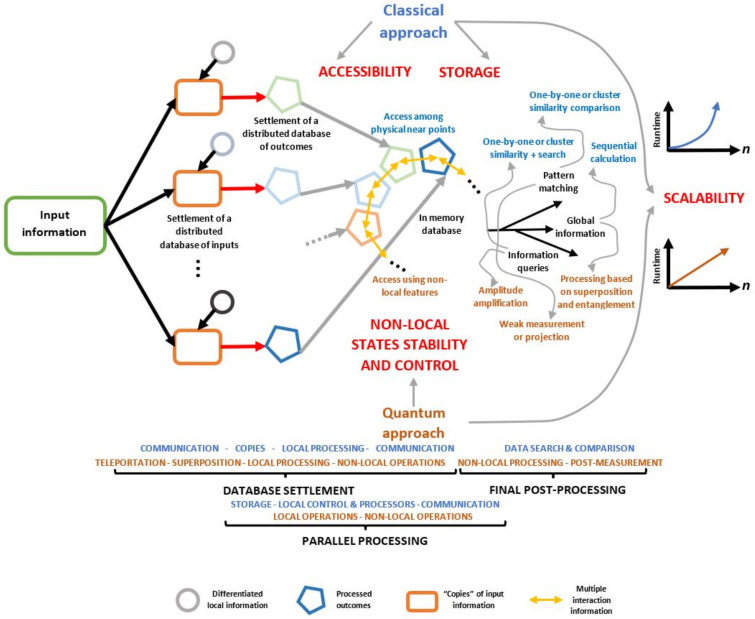
Classic (blue) and Quantum (brown) interactions between database settlement and parallel processing to first set a distributed database moved on one localized, temporary memory instance. Critical aspects for each approach are remarked in red.

Figure 1 shows a diagram summarizing the interactions between database settlement and specialized parallel processing. Departing from possible input information, several independent computing processes obtain differentiated derived results as output. Extensive problems fit such a process. For instance, the generation of alternative prototypes solves certain technological necessities, the determination of an optimized distribution path in logistics, or the decision making for the brain realizes how driving certain objects as the first experience departs from its perceived form and size. All those problems require diversified, prospective, and alternative views or solutions to be matched with a certain specific outcome or otherwise to obtain an integrated outcome comprising all individual solutions. Thus, Figure 1 compares the classical (blue) with the quantum (brown) approaches to both problems and their integration. Critical aspects highlighted in red for each approach are important functions of the scalability of concrete computing. Classical physical computing devices devote specific spaces to set each part of information. Thus, it requests storage along with speedy accessibility limited by distances and the growing number of interconnections among those parts, still considering an in-memory database processing [33].

Nonetheless, a quantum approach requires a tight control over the stability of quantum resources, particularly those exhibiting non-local properties. However, such limitations are superseded with the increasing development of quantum technologies, whereas classical systems exhibit strict final physical limitations owing to their nature. The settlement of information on classical devices requires centralized control to manage the storage increasing runtime upon scalability. It implies normally an exponential growth in the runtime for classical processing approaches, whereas in the quantum case, the growth is linear or polynomial. The same is true during parallel processing that still considers distributed processors with independent management systems. Quantum systems are less dependent on the storage space, not only due to the nature of systems employed, but also because they exhibit compact coding along with a natural processing speed up mainly due to superposition [34].

### 2.4. A Quantum Model for Database Distribution and Settlement

In the current development, a quantum database settlement (the initial multipartite storage of detonating information) will be developed by first teleporting a single qubit state (as instance, but easily generalized for bigger information states) on a set of potential receivers or processors via controlled multiple teleportation. Subsequently, such a set of processors will state a distributed database with an index naturally introduced by the control state previously used. Those processors can then introduce additional local information. Processors activate quantum parallelism by performing a series of differentiated processing tasks boosted by the original qubit. Accordingly, a partial selection of outcomes works as a filter. At the end, final global processing or matching tasks can be performed to extract or gain certain global information or otherwise to perform a pattern matching operation.

**Figure 2:**
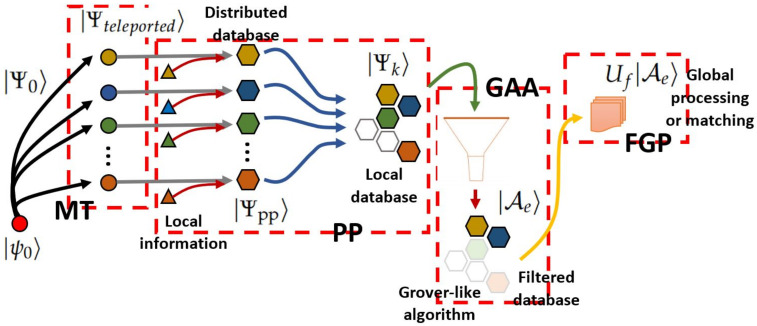
General scheme of the procedure with the steps considered: (MT, PP, GAA, and FGP). Each state mentioned is the final state of each step. Note that local information can be integrated in each local processing in the PP step.

In this study, the aforementioned procedure undergoes several structured steps: Multiple Teleportation (MT) and Post-processing (PP) developed in Section 3. In Section 4, the Grover’s Amplitude Amplification (GAA) for the current analysis is presented and analyzed. Finally, Section 5 presents suggestions and analysis for a Final Global Processing (FGP) or Pattern Matching (PM). Table 1 comprises several states, operators, and related quantities used in each main step mentioned. In addition, Figure 2 provides a previous summarized view of the entire process in terms of those elements.

## 3. Multiple Quantum State Distribution Model for the Database Settlement Based on Teleportation

The settlement of database is commonly supposed to be loaded or stated in many developments of quantum processing, pattern matching, or query problems. Reality is far different because setting ordered information on certain physical storage requires precise read and write operations [35]. Despite the complexity of managing non-local resources or efficient methods to perform teleportation, it can be a straightforward process for such a settlement, which nevertheless requires preparation to set entangled pairs in suitable locations. Current technological developments are successful, such a problem based on entanglement distribution. For instance, satellite-based entanglement distribution [36] ranges such delivery around 1200 km. Otherwise, state teleportation can naturally occur over short distances as molecular or biochemical structures at nanoscales [37,38].

Multiple teleportation process enables transmitting an arbitrary and possibly unknown quantum state. Particularly, double teleportation has been already exploited for cryptography purposes in the settlement of secure authentication [23] and in Quantum Key Distribution (QKD) [39]. Nonetheless, the double teleportation process has exhibited non-locality activation in the quantum state transference [40]. As an extension, in multiple teleportation, a state is quantumly transmitted in superposition to several receivers supported by other quantum states used as a control. Such receivers perform differentiated local processing operations stating a distributed database in superposition, but still identified by an index being introduced by the control state. The database is finally collected on one of those receivers.

**Figure 3:**
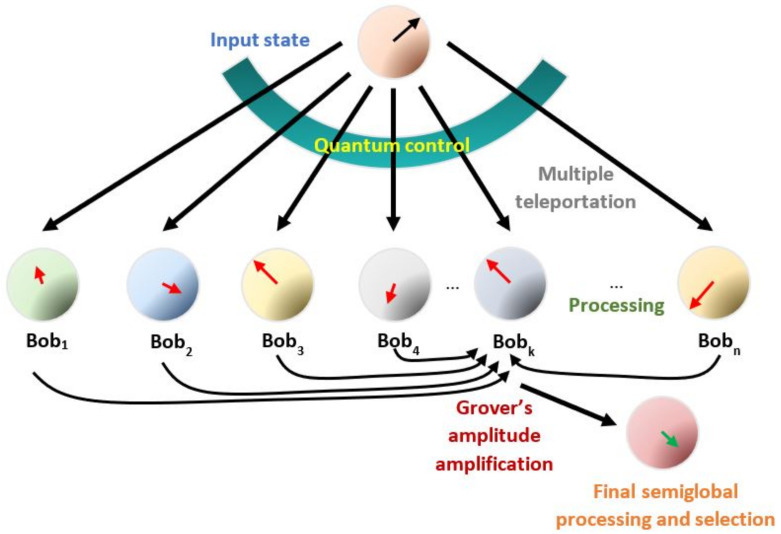
Further processing is performed by each receiver, subsequently translating the outcomes on a selected party, thereby setting a local database. Finally, a final processing or pattern matching can be performed using a subset of selected outcomes, either stochastically via measurement or using Grover’s selection.

By introducing the traditional characters of communication in the procedure, the original qubit whose state is teleported is originally in possession of Alice (the initial producer of detonating information), who has previously distributed a set of shared entangled resources on the receivers (which is called Bob’s; each one is the main part of each distributed processor), as in the traditional teleportation algorithm. Furthermore, the control state assists the process (it can also be in possession of Alice or another party) to possibly “decide” the final teleported state (i.e., superposition). In our approach, the original qubit is transferred to one specific party, and we independently process each distributed state to generate diversified information, possibly introducing additional local information: a distributed database. Finally, such outcomes are transferred on one party setting a local database in superposition to post-selecting a subset for further purposes. The process is depicted on Figure 3. Thus, the entire process beginning with multiple teleportation undergoes several processing layers: MT→PP→GAA→FGP.

### 3.1. Multiple Quantum Teleportation Procedure and Their Local and Non-Local Control: Dataset Settlement and Parallel Processing

The current process of teleportation (MT) begins with Alice attempting to teleport the state confined on a qubit labelled as 0: $|\varphi_{0}\rangle =\alpha_{0}{|0\rangle }_{0}+\alpha_{1}{|1\rangle }_{0}$. In fact, our process can be easily generalized to teleport larger quantum states $|\varphi_{0}\rangle$, at least those obtained as a combination of *p* two-level systems with dimension $m=2^{p},p\in Z$ [22]. It does not require deeper changes (particularly after the teleportation) than introducing additional entangled resources. We consider this simpler case to develop our procedure. The qubit is pretended to be potentially teleported on a group of *n* receivers called Bob${}_{1}$, Bob${}_{2}$, …, Bob${}_{n}$. For this task, she prepares a group of Bell entangled resources $|\beta_{00}\rangle_{2i-1,2i}=\frac{1}{\sqrt{2}}{(|00\rangle }_{2i-1,2i}+{|11\rangle }_{2i-1,2i}),i=1,2,\ldots ,n$, on qubits 1,2,…,2n. In addition, she shares one qubit of such resources sequentially, with each Bob${}_{i}$. In addition, someone prepares a control qubit with n−levels. Otherwise, it can be prepared using a set of *q* qubits to compose a state of dimension $n=2^{q},q\in Z$, as discussed later. Thus:

(1) $$\begin{array}{c} |\varphi_{C}\rangle =\sum_{i=1}^{n}\sqrt{p_{i}}\;{|i\rangle }_{C},\;\mathrm{with}:\;\sum_{i=1}^{n}p_{i}=1 \end{array}$$

then, the beginning total state to be considered can be expressed as:

(2) $$\begin{array}{c} |\Psi_{0}\rangle ={(\alpha_{0}|0\rangle }_{0}+\alpha_{1}{|1\rangle }_{0})⊗{(\sum_{i=1}^{n}\sqrt{p_{i}}\;|i\rangle }_{C})⊗\overset{n}{\underset{j=1}{⨂}}{|\beta_{00}\rangle }_{2j-1,2j} \end{array}$$

**Figure 4:**
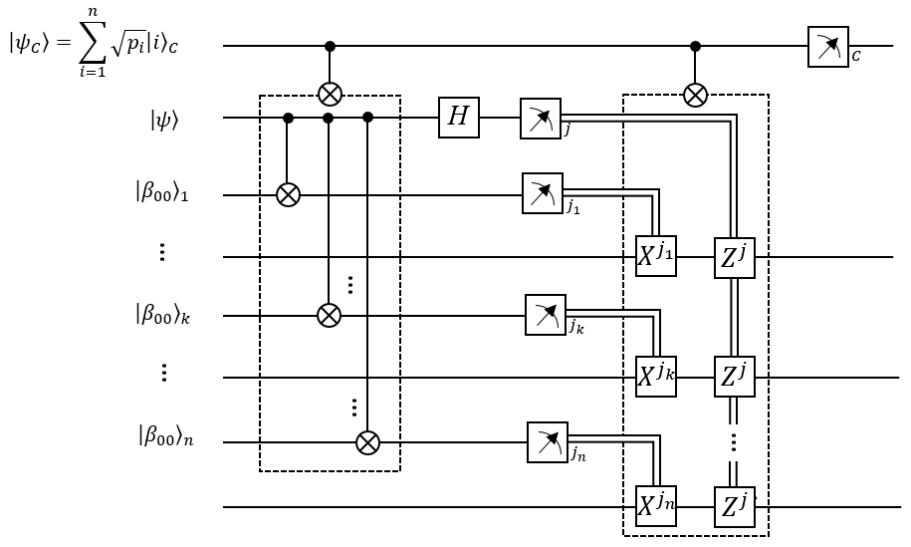
Quantum circuit version for multiple teleportation algorithm including a configurable qubit for their control $|\varphi_{C}\rangle$. A possible ending measurement on the control qubit with outcome |c〉 will definitively teleport the state |ψ〉 in the qubit 0 on the qubit 2c. Otherwise, teleportation remains superpositioned on the even qubits.

We consider an easier case as an illustrative approach using a two-level system as a detonating state. All our development can be extended by considering $|\varphi_{0}\rangle$ as a larger state than a qubit, or otherwise a composed state of two-level systems. In any case, it can also be teleported in the worst case, system by system, using the traditional teleportation algorithm with additional entangled resources [21,22], or more efficient algorithms using larger systems [41]. For the current approach, Figure 4 shows the entire quantum circuit of the process. Alice (or someone else including the management of the control state and the Alice state) prepares the following controlled gate to process her qubits, with the teleported qubit as control in this step:

(3) $$\begin{array}{c} C-CNOT^{n}\equiv \sum_{i=1}^{n}{|i\rangle }_{C}\langle i|⊗C^{0}NOT_{2i-1} \end{array}$$

here, $C^{a}U_{b}$ denotes the application of gate *U* on the qubit *b* controlled by the qubit *a*: $C^{a}U_{b}={|0\rangle }_{a}\langle 0|⊗1_{b}+{|1\rangle }_{a}\langle 1|⊗U_{b}$. The nature of this non-local gate and other included in the development will be discussed below. In fact, such a gate can be understood as a series of Toffoli-like gates $T_{2i-1}^{C,0}$:

(4) $$\begin{array}{c} C-CNOT^{n}=\prod_{i=1}^{n}T_{2i-1}^{C,0}\equiv \prod_{i=1}^{n}\left({|i\rangle }_{C}\langle i|⊗C^{0}NOT_{2i-1}+\sum_{i\neq j=1}^{n}{|j\rangle }_{C}\langle j|⊗1_{0}⊗1_{2i-1}\right) \end{array}$$

Clearly, such a gate is unitary if we reduce it by pairs: $\left(C-CNOT^{n}\right){\left(C-CNOT^{n}\right)}^{†}=1_{C}⊗1_{0,1,2,\ldots ,2n}$, where $1_{0,1,2,\ldots ,2n}$ is the identity in the subspace of the whole Bell entangled resources plus the input state. Thus, applying that gate, she obtains:

(5) $$\begin{array}{ccc} C-CNOT^{n}|\Psi_{0}\rangle & = & \sum_{i=1}^{n}\sqrt{p_{i}}{\;|i\rangle }_{C}⊗C^{0}NOT_{2i-1}{(\alpha_{0}|0\rangle }_{0}+\alpha_{1}{|1\rangle }_{0})⊗\overset{n}{\underset{j=1}{⨂}}{|\beta_{00}\rangle }_{2j-1,2j}\\ & = & \sum_{i=1}^{n}\sqrt{p_{i}}{\;|i\rangle }_{C}⊗(\alpha_{0}{|0\rangle }_{0}⊗\overset{n}{\underset{j=1}{⨂}}{|\beta_{00}\rangle }_{2j-1,2j}\\ & & \;+\alpha_{1}{|1\rangle }_{0}⊗\overset{n}{\underset{i\neq j=1}{⨂}}|\beta_{00}\rangle_{2j-1,2j}⊗X_{2i-1}{|\beta_{00}\rangle }_{2i-1,2i}) \end{array}$$

there, $X_{2i-1}$ is the NOT gate applied to the qubit 2i−1 inherited from $C^{0}NOT_{2i-1}$ (in general, X,Y and Z are the Pauli operators). By noting that $X_{2i-1}|\beta_{00}\rangle_{2i-1,2i}=|\beta_{01}\rangle_{2i-1,2i}=\frac{1}{\sqrt{2}}{(|01\rangle }_{2i-1,2i}+{|10\rangle }_{2i-1,2i})$ and:

(6) $$\begin{array}{ccc} \overset{n}{\underset{j=1}{⨂}}{|\beta_{00}\rangle }_{2j-1,2j} & = & \overset{n}{\underset{j=1}{⨂}}\frac{1}{\sqrt{2}}\left({|00\rangle }_{2j-1,2j}+{|11\rangle }_{2j-1,2j}\right)\\ & = & \frac{1}{{\sqrt{2}}^{n}}\sum_{s=0}^{N}{|s\rangle }_{\mathrm{odd}}⊗{|s\rangle }_{\mathrm{even}} \end{array}$$

there, $N=2^{n}-1$. Additionally, *odd* and *even* refer to a set of qubits with labels odd or even, respectively, conforming two composed n−partite qubits ${|s\rangle }_{\mathrm{even}}$ and ${|s\rangle }_{\mathrm{odd}}$ labelled with the 10−base number *s* whose 2−base digits set the states of qubits conforming them. Thus, upon the application of $C-CNOT^{n}$, the expansion provides all possible combinations of 0’s and 1’s for each qubit labelled with odd and even numbers by separate, but paired, with exception for the qubit *i* in the second term owing to the NOT gate. Thus, the global state becomes:

(7) $$\begin{array}{ccc} C-CNOT^{n}|\Psi_{0}\rangle & = & \sum_{i=1}^{n}\frac{\sqrt{p_{i}}}{{\sqrt{2}}^{n}}{\;|i\rangle }_{C}⊗(\alpha_{0}{|0\rangle }_{0}⊗\sum_{s=0}^{N}{|s\rangle }_{\mathrm{odd}}⊗{|s\rangle }_{\mathrm{even}}\\ & & \;+\alpha_{1}{|1\rangle }_{0}⊗\sum_{s=0}^{N}{|s\rangle }_{\mathrm{odd}}⊗{|s+{\left(-1\right)}^{s_{i}^{o}}2^{i-1}\rangle }_{\mathrm{even}}) \end{array}$$

In such an expression, $s_{i}^{o}$ is the *i*-th digit in *s* when it is expressed in 2-base. Accordingly, Alice follows the teleportation process by applying a Hadamard gate on the qubit 0, thus obtaining the following:

(8) $$\begin{array}{ccc} H_{0}\cdot C-CNOT^{n}|\Psi_{0}\rangle & = & \sum_{i=1}^{n}\frac{\sqrt{p_{i}}}{{\sqrt{2}}^{n+1}}{\;|i\rangle }_{C}⊗(\alpha_{0}{(|0\rangle }_{0}+{|1\rangle }_{0})⊗\sum_{s=0}^{N}{|s\rangle }_{\mathrm{odd}}⊗{|s\rangle }_{\mathrm{even}}\\ & & \;+\alpha_{1}{(|0\rangle }_{0}-{|1\rangle }_{0})⊗\sum_{s=0}^{N}{|s\rangle }_{\mathrm{odd}}⊗{|s+{\left(-1\right)}^{s_{i}^{o}}2^{i-1}\rangle }_{\mathrm{even}}) \end{array}$$

After, Alice performs a measurement of qubits 0,1,3,…,2n−1 obtaining the outcomes $j,s_{1},s_{2},\ldots ,s_{n}$, respectively (last outcomes corresponds to the 2−base representation of the 10−base number *s*). Thus, the post-measurement state becomes:

(9) $$\begin{array}{ccc} |\Psi_{\mathrm{pm}}\rangle & = & \sum_{i=1}^{n}\sqrt{p_{i}}{\;|i\rangle }_{C}⊗{|j\rangle }_{0}⊗{|s\rangle }_{\mathrm{odd}}⊗\left(\alpha_{0}{|s\rangle }_{\mathrm{even}}+{\left(-1\right)}^{j}\alpha_{1}{|s+{\left(-1\right)}^{s_{i}^{o}}2^{i-1}\rangle }_{\mathrm{even}}\right)\\ & = & \sum_{i=1}^{n}\sqrt{p_{i}}{\;|i\rangle }_{C}⊗{|j\rangle }_{0}⊗{|s\rangle }_{\mathrm{odd}}⊗{|s\rangle }_{\mathrm{even}}^{\left\{2i\right\}}⊗\left(\alpha_{0}|s_{i}^{o}\rangle_{2i}+{\left(-1\right)}^{j}\alpha_{1}{|s_{i}^{o}+{\left(-1\right)}^{s_{i}^{o}}2^{i-1}\rangle }_{2i}\right)\\ & = & \sum_{i=1}^{n}\sqrt{p_{i}}{\;|i\rangle }_{C}⊗{|j\rangle }_{0}⊗{|s\rangle }_{\mathrm{odd}}⊗{|s\rangle }_{\mathrm{even}}^{\left\{2i\right\}}⊗X_{2i}^{s_{i}}Z_{2i}^{j}{|\varphi_{0}\rangle }_{2i} \end{array}$$

where ${|s\rangle }_{\mathrm{even}}^{\left\{2i\right\}}$ refers to the entire n−1 even qubits in the state ${|s\rangle }_{\mathrm{even}}$ except by the 2i qubit. Alice uses classical communication to share the outcomes with each Bob${}_{k}$. Thus, they apply the following controlled operations on their qubits (see Figure 4):

(10) $$\begin{array}{c} C_{{\mathrm{Bob}}^{\prime }s}\equiv \sum_{i=1}^{n}{|i\rangle }_{C}\langle i|⊗Z_{2i}^{j}⊗\overset{n}{\underset{k=1}{⨂}}X_{2k}^{s_{k}} \end{array}$$

which means they take the input of measurements, but they are controlled by system *C*. Such correction transforms 1’s into 0’s for all qubits, with exception of qubit *i* (in each term of the sum):

(11) $$\begin{array}{ccc} C_{{\mathrm{Bob}}^{\prime }s}\cdot |\Psi_{\mathrm{pm}}\rangle & = & \sum_{i=1}^{n}\sqrt{p_{i}}{\;|i\rangle }_{C}⊗{|j\rangle }_{0}⊗{|s\rangle }_{\mathrm{odd}}⊗{|0\rangle }_{\mathrm{even}}^{⊗n\backslash \{2i\}}⊗\left(\alpha_{0}{|0\rangle }_{2i}+\alpha_{1}{|1\rangle }_{2i}\right) \end{array}$$

where ${|0\rangle }_{\mathrm{even}}^{⊗n\backslash \{2i\}}={⨂}_{i\neq k=1}^{n}X_{2k}^{s_{k}}{|s\rangle }_{\mathrm{even}}^{\left\{2i\right\}}$ refers to the entire n−1 even qubits in the state |0〉, with exception of 2i. Note that, independently to the outcomes in the measurements, we arrive to the state:

(12) $$\begin{array}{ccc} |\Psi_{\mathrm{teleported}}\rangle & = & \sum_{i=1}^{n}\sqrt{p_{i}}{\;|i\rangle }_{C}⊗{|0\rangle }_{\mathrm{even}}^{⊗n\backslash \{2i\}}⊗\left(\alpha_{0}{|0\rangle }_{2i}+\alpha_{1}{|1\rangle }_{2i}\right) \end{array}$$

for the control and the even qubits by disregarding the remaining qubits. Finally, if the control qubit is measured with the outcome |i〉 (which has probability $p_{i}$), the information of the original qubit 0 is teleported to Bob${}_{i}$. The quantum circuit corresponding to the entire process has been graphically represented in Figure 4. This includes an alternative ending, post measurement of the control state, which will be discussed below.

### 3.2. Multiple Semi-Local Parallel Processing to State a Local Database of Outcomes and Their Coherent Gathering: In-Memory Post-Processing

Notably, Bob knows who is in possession of the teleported qubit. Nonetheless, Alice cannot be sure of who will receive the teleported qubit; regardless, she initially prepares the control state to favor one or several selected qubits of Bob (if she has the control qubit). Formula (12) implies that as a quantum superposition, the information on qubit 0 has been potentially teleported to Bob. Moreover, as illustrated in Figure 3, quantum parallelism is activated by Bob by performing a different controlled quantum processing $U_{i_{2i}}$ on the qubit 2i based on the control state, considering $\alpha_{0}{|0\rangle }_{2i}+\alpha_{1}{|1\rangle }_{2i}$ as input:

(13) $$\begin{array}{c} C-U\equiv \sum_{i=1}^{n}{|i\rangle }_{C}\langle i|⊗1_{\mathrm{even}}^{⊗n\backslash \{2i\}}⊗U_{i_{2i}} \end{array}$$

**Figure 5:**
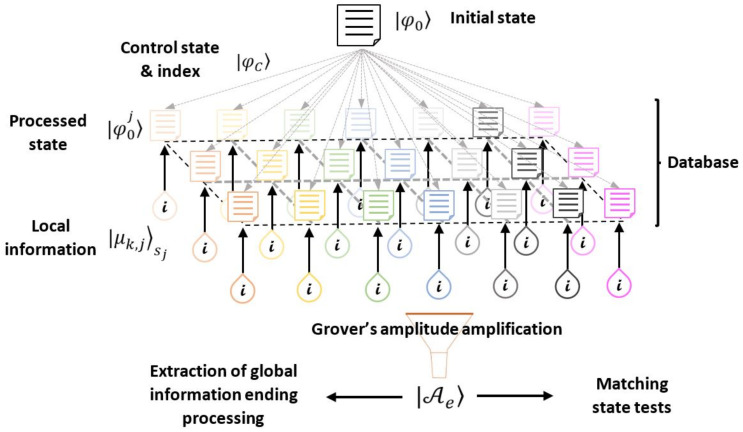
Settlement of a database using multiple teleportation and post-processing. It is based on a detonating initial state on local receivers integrating local information and processing. A selection of some outcomes can be obtained using amplitude amplification. The final output state can be used to extract global information or to match an external state on the database.

This step sets the parallel processing (PP). Accordingly, if $|\varphi_{0}^{i}\rangle_{2i}\equiv \alpha_{0}^{i}{|0\rangle }_{2i}+\alpha_{1}^{i}{|1\rangle }_{2i}$ is the output of each processing $U_{i_{2i}}$. Thus, we obtain:

(14) $$\begin{array}{ccc} |\Psi_{\mathrm{pp}}\rangle \equiv C-U|\Psi_{\mathrm{teleported}}\rangle & = & \sum_{i=1}^{n}\sqrt{p_{i}}{\;|i\rangle }_{C}⊗{|0\rangle }_{\mathrm{even}}^{⊗n\backslash \{2i\}}⊗{|\varphi_{0}^{i}\rangle }_{2i} \end{array}$$

which potentially comprises all outcomes of the set of processing $U_{i}$. Concretely, it sets the distributed database being indexed by ${|i\rangle }_{C}$. Additionally, all such outcomes can be transferred to Bob (see Figure 3), namely Bob${}_{k}$, by means of a controlled SWAP gate (SWAP gate exchanges the states between qubits *a* and *b* as $SWAP_{a,b}=\sum_{i,j=0}^{1}{|i\rangle }_{a}\langle j|⊗{|j\rangle }_{b}\langle i|$) in the form:

(15) $$\begin{array}{c} C-SWAP\equiv \sum_{i=1}^{n}{|i\rangle }_{C}\langle i|⊗SWAP_{2i,2k} \end{array}$$

thus obtaining the following state separated from the remaining Bobs (omitting the tensor product symbols for simplicity):

(16) $$\begin{array}{c} |\Psi_{k}{\rangle ⊗|0\rangle }_{\mathrm{even}}^{⊗n\backslash \{2k\}}\equiv C-SWAP|\Psi_{\mathrm{pp}}\rangle =\left(\sum_{i=1}^{n}\sqrt{p_{i}}{|i\rangle }_{C}⊗{|\varphi_{0}^{i}\rangle }_{2k}\right){|0\rangle }_{\mathrm{even}}^{⊗n\backslash \{2k\}} \end{array}$$

with $|\varphi_{0}^{i}\rangle_{2k}\equiv \alpha_{0}^{i}{|0\rangle }_{2k}+\alpha_{1}^{i}{|1\rangle }_{2k}$. Although Alice cannot ensure in general the definitive post-delivering to Bob, she manipulates the control probabilities $p_{i}$. Otherwise, the collaboration among Bobs is feasible, if they have access to the control qubit and $U_{i}$ is not still applied. Accordingly, the last action sends the teleported state to Bob${}_{k}$, whose state becomes separable from the remaining system state. In any case, $|\Psi_{k}\rangle$ can be understood as a local database of the processing outcomes distributed across several parties with an index provided by the control state. At this point, it should be remembered that $|\varphi_{0}\rangle$ can be the most complex multipartite quantum state; accordingly each $|\varphi_{0}^{i}\rangle_{2k}$, almost following the same development. Nevertheless, in the immediate following development, we maintain the single qubit character for each processed qubit, although it can be addressed as a more complex entity by including the local information in each local processing as it will be discussed in Section 5. They are additionally indexed by the control state ${|i\rangle }_{C}$. The global scheme of the process is shown in Figure 5. We will address the complementary selection of outcomes via measurement or based on Grover’s amplitude amplification, to finally use the output state to obtain global information from the parallel processing or to match with certain external state of the database.

Thus, despite the no-cloning theorem, it is still possible to reproduce certain arbitrary and possibly unknown state to enable parallel processing to obtain a convenient linear combination of the outcomes. Still, some criticism can be made of the operations performed among supposed faraway qubits as the control one (*C*) and those of each Bob and among Bobs. Despite such operations expressed synthetically before, developments in terms of local operations with classical communication (LOCC) are noted. Thus, operations as C−U and C−SWAP are suitably developed in Appendices Appendix A (at least for $n=2^{p},p\in Z$, which still fits our interests) and Appendix B, respectively. In the next section, we will analyze how to select a subset of such outcomes from the database via an adaptation of Grover algorithm.

## 4. Search and Selection of Processed States on the Database Gathering

In the current section, we deal with the post-selection of a subset of processing outcomes in the local database emerging from the previous procedure. This task is completed by stochastic post-selection or otherwise through a modification of Grover’s algorithm, considering bipartite states. For the last procedure, those states still become orthogonal because the indexing stated from the control states and their properties.

### 4.1. Stochastic Post-Selection on the Local Database

Otherwise, as part of the information extraction from the state in (16), Alice can attempt another measurement different from that performed on the original control basis ${|i\rangle }_{C}$ by changing the indexing and mixing the single post-processing. For instance, on a set $|c_{l}\rangle_{C}=\sum_{i=1}^{n}\gamma_{i}^{l}{|i\rangle }_{C}$ (in the following, we restrict our analysis to $\gamma_{i}^{l}\in R$). It immediately projects on Bob${}_{k}$, a state with certain *l*-linear combination of the entire information involved with the output states arising from the different processes, as a possible additional step of the information extraction:

(17) $$\begin{array}{c} |\Psi_{{\mathrm{Bob}}_{k}}^{l}\rangle \equiv \frac{1}{\sqrt{P_{c_{l}}}}\langle c_{l}|\Psi_{k}\rangle =(\sum_{i=1}^{n}\gamma_{i}^{l}\sqrt{\frac{p_{i}}{P_{c_{l}}}}U_{i_{2i}})|\varphi_{0}\rangle \end{array}$$

This state corresponds to Bob${}_{k}$. Such stochastic selection can restrict the combinations of information selectively, although the probabilities of success to obtain certain outcomes become small if the class they belong to is a small part of the entire set $\left\{|\varphi_{0}^{i}\rangle \right\}$:

(18) $$\begin{array}{ccc} P_{c_{l}} & = & |\langle c_{l}|\Psi_{k}\rangle {|}^{2}=\sum_{i,j=1}^{n}\gamma_{i}^{l}\gamma_{j}^{l}\sqrt{p_{i}p_{j}}\langle \varphi_{0}^{i}|\varphi_{0}^{j}\rangle \\ & = & \langle \varphi_{0}|(\sum_{i=1}^{n}\gamma_{i}\sqrt{p_{i}}U_{i_{2i}}{)}^{†}(\sum_{j=1}^{n}\gamma_{j}\sqrt{p_{j}}U_{j_{2j}})|\varphi_{0}\rangle \end{array}$$

Such a set of measurements on a different basis for the control state was used in QKD to distribute a key on a secure channel through double teleportation [23,39]. In addition, constructing a physical measurement specifically containing the desired outcomes as one of the measurement basis elements is necessary. Although stochastic post-selection is a relevant quantum information resource reproduced artificially through a measurement device on a physical quantum system, the possibility that the brain will be able to perform self-measurement (at least as a decoherence phenomenon) has opened up discussions into quantum cognition regarding the quantum properties of consciousness [42].

Alternative stochastic methods could be provided by weak measurements. For instance, if certain subset S of outcomes $\left\{|\varphi_{0}^{i}\rangle |i\in S\right\}$ is searched, then the pair of weak measurements can be attempted considering the measurement operators:

(19) $$\begin{array}{ccc} W_{0} & = & |\Psi_{S}\rangle \langle \Psi_{S}| \end{array}$$

(20) $$\begin{array}{ccc} W_{1} & = & 1-W_{0} \end{array}$$

(21) $$\begin{array}{ccc} & & \mathrm{with}:\;|\Psi_{S}\rangle =\frac{1}{\sqrt{N_{S}}}\sum_{i\in S}|\varphi_{0}^{i}\rangle \end{array}$$

being $N_{S}$ the cardinality of S. Such alternative functions appropriately when states $|\varphi_{0}^{i}\rangle$ are orthogonal.

Alternatively, we can reduce such sets by using an amplitude amplification algorithm [24], thus delivering a more selected subset from the entire outcomes in the database. Furthermore, a possible post-measurement of the control can still stochastically deliver a more specific desired combination from the previous amplitude amplification. The amplitude amplification algorithm for our concrete state (17) can be analyzed and developed in the next section.

### 4.2. Coherent Post-Selection Using a Controlled Amplitude Amplification

Similar types of Grover-like algorithm have been proposed to deal with image pattern matching [43] by the Approximate Amplitude Encoding (AAE) method to set the database. In the current development, multiple teleportation and post-processing have been used to set a database of codified quantum states possibly gathering local information incoming from several locations.

Thus, we afford a soft controlled and coherent post-selection of outcomes incoming from the previous procedure by a Grover-like method for amplitude amplification, the third step in the procedure (GAA). In the next sections, recursive formulas for the processed state in each Grover-like algorithm step are obtained and reported and an associated analysis for the effectiveness of the method under several possible scenarios is conducted.

#### 4.2.1. Quantum Search and Single Selection on the Local Database

Considering the state $|\Psi_{k}\rangle =\sum_{i=1}^{n}\alpha_{i}|A_{i}\rangle$ in (16) with $|A_{i}{\rangle =|i\rangle }_{C}⊗|\varphi_{0}^{i}\rangle ,\alpha_{i}=\sqrt{p_{i}}$ (disregarding the label corresponding to Bob${}_{k}$). It can be considered to virtually contain the information of the *n*-parallel calculation processing. Probably the most typical application for the current procedure is the extraction of a certain outcome $|\varphi_{0}^{k}\rangle$ from the entire database when required. Alice can use the Grover algorithm [24] to increase its corresponding amplitude $|\alpha_{k}{|}^{2}$ to one. The following unitary operators are required: an Oracle $U_{e}$, and the Grover diffusion operator $U_{m}$.

(22) $$\begin{array}{ccc} U_{e} & = & 1-2|A_{e}\rangle \langle A_{e}\left|,\;\mathrm{with}:\;\right|A_{e}\rangle =|A_{k}\rangle \end{array}$$

(23) $$\begin{array}{ccc} U_{m} & = & 2|\Psi^{\left(0\right)}\rangle \langle \Psi^{\left(0\right)}\left|-1,\;\mathrm{with}:\;\right|\Psi^{\left(0\right)}\rangle =\frac{1}{\sqrt{n}}\sum_{i=1}^{n}|A_{i}\rangle \end{array}$$

Thus, by applying the Grover algorithm repetitively $|\Psi^{\left(s\right)}\rangle =U_{m}U_{e}|\Psi^{\left(s-1\right)}\rangle$ on $|\Psi^{\left(s\right)}\rangle =\sum_{i=1}^{n}\alpha_{i}^{\left(s\right)}|A_{i}\rangle ,s=0,1,2,\ldots ,R\in N$ (with: $\alpha_{i}^{\left(0\right)}\equiv \alpha_{i}$). As a direct calculation after applying $U_{m}U_{e}$ in each step, the following iterative relations are obtained:

(24) $$\begin{array}{ccc} \alpha_{k}^{\left(s\right)} & = & \frac{2}{n}\sum_{l=1}^{n}\alpha_{l}^{\left(s-1\right)}+\left(1-\frac{4}{n}\right)\alpha_{k}^{\left(s-1\right)} \end{array}$$

(25) $$\begin{array}{ccc} \alpha_{j\neq k}^{\left(s\right)} & = & \alpha_{k}^{\left(s\right)}-\left(\alpha_{k}^{\left(s-1\right)}+\alpha_{j}^{\left(s-1\right)}\right),\;s=1,2,\ldots ,R \end{array}$$

**Figure 6:**
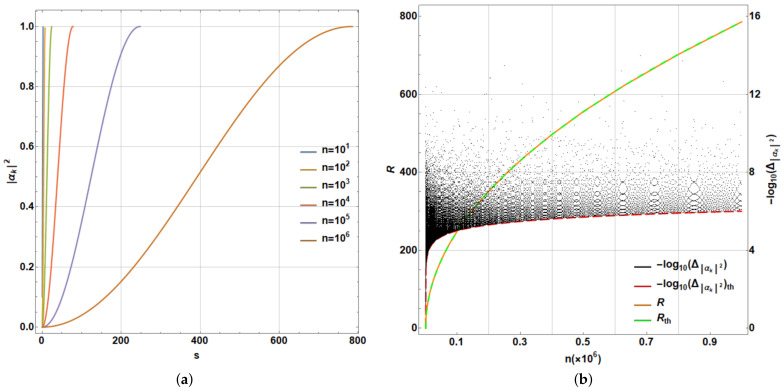
(**a**) Repetitions *s* of Grover algorithm to extract the processing $|\varphi_{0}^{k}\rangle$ raising its amplitude $|\alpha_{k}{|}^{2}$; (**b**) Number of repetitions *R* (orange dashed line) to raise $|\alpha_{k}{|}^{2}\approx 1$, along with its associated logarithmic error $-\log(\Delta_{|\alpha_{k}{|}^{2}})$ (black dots), compared with the theoretical values (red dashed line).

It is widely known that the necessary number of repetitions to raise the amplitude $|\alpha_{k}^{\left(0\right)}{|}^{2}$ to approximately one is $R\to \frac{\pi \sqrt{n}}{4}\equiv R_{\mathrm{th}}$ if n→∞ [24]. In addition, the least error to reach $|\alpha_{k}^{\left(0\right)}{|}^{2}\approx 1$, denoted by $\Delta_{|\alpha_{k}{|}^{2}}\equiv 1-{\left|\alpha_{k}^{\left(R\right)}\right|}^{2}\to \frac{1}{n}\equiv {\Delta_{|\alpha_{k}{|}^{2}}}_{\mathrm{th}}$ if n→∞ [24]. Figure 6 illustrates such a procedure. In Figure 6a, several iterative applications for $n=10^{1},10^{2},\ldots ,10^{6}$ are shown as functions of the number of iterations, *s*. Figure 6b exhibits the value of *R* (orange dashed line) as it is compared with its theoretical value $R_{\mathrm{th}}$ (solid green line). Together, $\Delta_{|\alpha_{k}{|}^{2}}$ (black dots) as function of *n*, note the irregular dispersion of $\Delta_{|\alpha_{k}{|}^{2}}$ while *n* changes. Furthermore, note the fulfilling of the maximum error threshold. By departing from $\left|\Psi \rangle =\right|\Psi^{\left(0\right)}\rangle$ ($\alpha_{i}^{\left(0\right)}\equiv \alpha_{i}=\sqrt{p_{i}}=\frac{1}{\sqrt{n}}$), the theoretical limit values $R_{\mathrm{th}}$ and ${\Delta_{|\alpha_{k}{|}^{2}}}_{\mathrm{th}}$ for n→∞ [24] are included (green solid and red dashed lines, respectively). $\Delta_{|\alpha_{k}{|}^{2}}$ is reported in a logarithmic scale on the right side, exhibiting a fast dropping of the worst error below $10^{-6}$.

#### 4.2.2. Multiple Selection on the Local Database: Precision and Efficiency in the Grover’s Search

**Figure 7:**
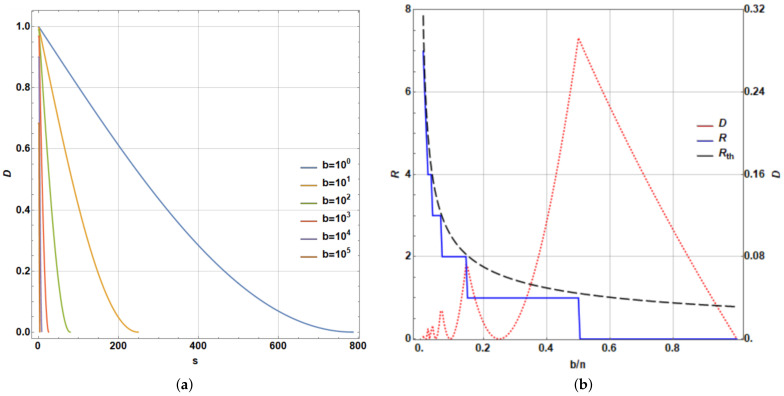
For $n=10^{6}$ and $|A_{e}\rangle =\frac{1}{\sqrt{b}}\sum_{k\in K}|A_{k}\rangle$: (**a**) Repetitions *s* and distance *D* evolution of Grover algorithm for several values of *b*; (**b**) Number of repetitions needed *R* (solid blue line) compared with the theoretical value $R_{\mathrm{th}}$ (dashed black line) along with *D* (dashed red line) as a function of $\frac{b}{n}$.

Although Alice performs this selection *a posteriori*, she could not be interested in the extraction of only one outcome (otherwise is better generate it directly), rather several such outcomes. For instance, if the desired target state is $|A_{e}\rangle =\sum_{k\in K}\beta_{k}|A_{k}\rangle$, with *K* a subset of labels defining a subspace of the entire Hilbert space generated by $\left\{|A_{k}\rangle |k\in K\right\}$, with cardinality b=C(K)≤n. The process for the Grover algorithm is followed, departing from the same initial state $|\Psi^{\left(0\right)}\rangle$. The corresponding recursive equations for $\alpha_{i}^{\left(s\right)}$ become:

(26) $$\begin{array}{ccc} \alpha_{k\in K}^{\left(s\right)} & = & \frac{2}{n}\left(\sum_{l\in J}\alpha_{l}^{\left(s-1\right)}+\sum_{k^{\prime }\in K}\gamma_{k^{\prime }}^{\left(s-1\right)}\right)-\gamma_{k}^{\left(s-1\right)} \end{array}$$

(27) $$\begin{array}{ccc} \alpha_{j\in J}^{\left(s\right)} & = & \frac{2}{n}\left(\sum_{l\in J}\alpha_{l}^{\left(s-1\right)}+\sum_{k^{\prime }\in K}\gamma_{k^{\prime }}^{\left(s-1\right)}\right)-\alpha_{j}^{\left(s-1\right)},\;s=1,2,\ldots ,R \end{array}$$

(28) $$\begin{array}{ccc} & & \mathrm{with}:\;\gamma_{k\in K}^{\left(s-1\right)}=\alpha_{k}^{\left(s-1\right)}-2\beta_{k}\sum_{k^{\prime }\in K}\alpha_{k^{\prime }}^{\left(s-1\right)}\beta_{k^{\prime }}^{*} \end{array}$$

being *J* the complement of *K* in {1,2,…,n}. A measure of success can be constructed based on the Cartesian norm: $D\equiv \left||\right|A_{e}\rangle -|\Psi^{\left(R\right)}{\rangle ||}_{K}$ calculated only on the goal subspace. Despite such restriction, owing to Grover algorithm involving only unitary transformations, this distance provides an adequate measurement of distance, which if converges to zero, will meet the overall distance calculated on the entire Hilbert space of the state being modified. In addition, it provides, for simulation purposes, a computational advantage in terms of time processing, particularly with larger states. To illustrate the last procedure, we consider $n=10^{6}$ and $|A_{e}\rangle =\frac{1}{\sqrt{b}}\sum_{k\in K}|A_{k}\rangle$. Thus, Figure 7a shows the evolution of $D_{k}\equiv \left||\right|A_{e}\rangle -|\Psi^{\left(k\right)}{\rangle ||}_{K}$ (an equivalent measure for $\Delta_{|\alpha_{k}{|}^{2}}^{1/2}$), while reaching *D* as function of the number of iterations *s* of Grover algorithm until *R* for certain values of *b*. Note the reduction in *R* with an increase in *b*, only denoting the impossibility to reach $|A_{e}\rangle$.

Still considering the last example, Figure 7b shows *R* and $R_{\mathrm{th}}=\frac{\pi }{4}\sqrt{\frac{n}{b}}$ [44] (solid blue and dashed black lines, respectively). On the same plot, *D* as function of $\frac{b}{n}$ (dashed red line) is exhibited on the right scale, noting limitations for the multiple search due to the increasing number of terms *b* involved in the target $|A_{e}\rangle$, with a maximum D≈0.2929 for $b=\frac{n}{2}$. It is noticeable that peaks in the *D* dependence, clearly coincide with the discontinuous jumps for the *R* values. In fact, it is easily noticeable that while larger values of $R_{\mathrm{th}}\in Z$ are reached, lowest peaks occur in *b*: $\frac{b}{n}\approx {\left(\frac{\pi }{4R_{\mathrm{th}}}\right)}^{2}$ for the largest values of $R_{\mathrm{th}}$. In addition, as the peaks occur barely on these values and the error in the Grover algorithm for multiple post-selection is $\frac{b}{n}\approx 2D$ [44], such peaks become $D\approx \frac{1}{2}{\left(\frac{\pi }{4R_{\mathrm{th}}}\right)}^{2}$, particularly for $R_{\mathrm{th}}\in Z$ large. Those values accurately fit those observed in Figure 7b, while the last integer values for $R_{\mathrm{th}}$ are larger.

Moreover, in addition to the last analysis for $n=10^{6}$, for other values of *n* where the behavior between *R* and $R_{\mathrm{th}}$ is similar, we can perform the corresponding analysis for the goodness of Grover algorithm. In fact, independent of *n*, the behavior between *D* versus $\frac{b}{n}$ provides an identical plot to that shown in the Figure 7b (dashed red line), using the range of $\frac{b}{n}\in \left(0,1\right)$. For this reason, we do not report additional plots, despite the fact that they were calculated for $10^{k},k=2,3,\ldots ,7$ providing an exact coincidence. This means that the performance measured by *D* does not depend on *n*, rather only on $\frac{b}{n}$. Clearly, a growing number of repetitions of the Grover’s algorithm depending on $\frac{\pi }{4}\sqrt{\frac{n}{b}}$ as the upper limit is necessary. The peak behavior observed in the red curve for *D* is stated by $D\approx \frac{1}{2}{\left(\frac{\pi }{4R_{\mathrm{th}}}\right)}^{2}$ with $R_{\mathrm{th}}\in Z$. For a larger $\frac{b}{n}$, Grover algorithm does not enable accurate fitting of the desired state because the output state of the algorithm is only one step away from it in the algorithm. When $\frac{b}{n}$ decreases, the algorithm provides better outcomes, still in the scale of $\frac{b}{n}≲0.1$, thus providing a region for the best performance of the current selection method.

#### 4.2.3. Analysis of Convergence in Multiple Selection for the Most Typical Scenarios

**Figure 8:**
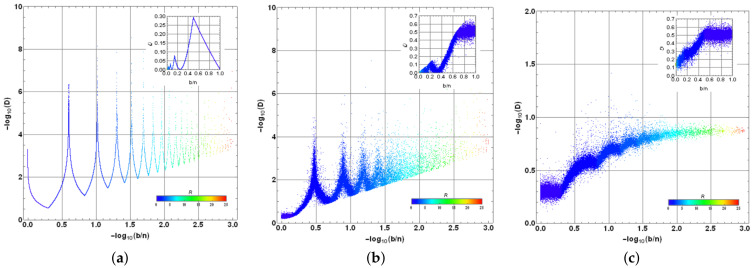
Distribution of distance *D* versus ratio $\frac{b}{n}$ in log-scales with the number of repetitions *R* in colour in agreement with the colour scale below, for: (**a**) both evenly distributed initial and target states, (**b**) evenly distributed initial state and randomly distributed target state, and (**c**) randomly distributed initial state and evenly distributed target state. Distributions were obtained by selecting $10\leq n\leq 10^{3}$ and 1≤b≤n. Upper-right insets show the normal scales in comparison with Figure 7.

We have attempted as examples the cases when both initial and target states are evenly distributed in their components ($\alpha_{i}=1/\sqrt{n}$ and $\beta_{k}=1/\sqrt{b}$). The behavior for other cases can be investigated in terms of convergence. Thus, by generating arbitrary states selected randomly with $10\leq n\leq 10^{3}$ and then 1≤b≤n, we analyze several cases by comparing $\frac{b}{n}$ and *D*. Figure 8a shows the previous case with initial and target states even. We are using log scales in both axes $\frac{b}{n}$ and *D* for a better appreciation of colors representing the necessary *R*. Thus, upper peaks represent the minimum values for *D*. Each dot corresponds to each case analyzed randomly on a total set of $10^{5}$ cases. The upper-right inset represents the same distribution with normal scales as in Figure 7b for comparison. Note that the same plot is obtained by gathering all values considered for *n* and *b* because the dependence is only on $\frac{b}{n}$, thus reproducing the same line. Similarly, Figure 8b exhibits the case where the target state is arbitrary and randomly generated. Note how the previous line transforms into a distribution due to the several values of *n*, *b* and the suitable target state. Nonetheless, the peaks indicating the previous minimum *D* values are preserved along with the general structure of Figure 8a, nonetheless with certain dispersion. Finally, Figure 8c shows the case where the target state is evenly distributed on its entries; however, the initial state is randomly selected. Such case shows a deeper deformation of the previous behavior with increased values for *D* and the dispersion introduced by several values for *n*, *b* along with the suitable initial state.

**Figure 9:**
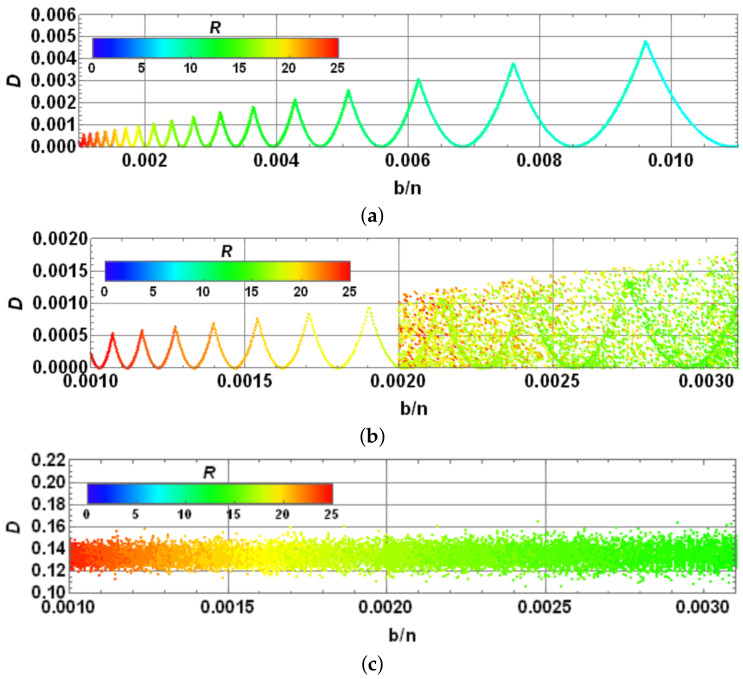
Close-up to plots in Figure 8 in the region b/n≪1 in normal scales for (**a**) evenly distributed initial and target states, (**b**) evenly distributed initial state and randomly distributed target state, and (**c**) randomly distributed initial state and evenly distributed target state.

Despite the last outcomes, the algorithm being analyzed functions suitably for the lowest values of b/n, if the initial state is evenly distributed. A close-up of those regions is shown in Figure 9 for the same cases previously reported (with normal scales): (a) evenly distributed initial and target states, (b) evenly distributed initial state and randomly distributed target states, and (c) randomly distributed initial state and evenly distributed target state. Figure 9a exhibits a sharp behavior for the evenly distributed scenarios. Note the behavior $\frac{b}{n}\approx 2D$ for the peaks. Figure 9b for the random target state exhibits a behavior still bounded as before, nonetheless dispersed. The transition from a line into a dispersion distribution is due $n\leq 10^{3}$; thus, in the range b/n∈[0.001,0.002], *b* has only a concrete possible value b=1, thus reducing to evenly target cases for the all possible values of *n*. For $\frac{b}{n}\geq 0.002$, we have the dispersion generated at least for the diverse target states (note the accumulation suggesting a remnant line), nonetheless still bounded. Figure 9c for the random initial states depicts a narrow distribution for *D* but is not precisely low. Such outcomes highlight the importance to begin with an evenly distributed state for the amplitude amplification problem being analyzed, which still provides suitable outcomes for b≪n cases.

At this point, we have performed the creation of a distributed database possibly boosted by a detonating state carrying initial information to be diversified integrating local information incoming from each processor. By gathering a single party, we have performed a partial post-selection using a Grover-like amplitude amplification algorithm. In the development, we focus on post-selection, still be composed by several outcomes incoming from the entire database. Because a possible further application of pattern matching can be performed, for the sake of generality, we assume that the first post-selection containing several outcomes has been performed, instead of a direct Grover search containing a single state to be matched (nonetheless, such a direct scenario is also possible). Grover’s amplitude amplification algorithm appears more artificial than the stochastic post-selection approach (at least considered as a decoherence process) to be present in natural processes. Nevertheless, there is certain evidence that eukaryotic cells perform natural quadratic speed-up in the search for an appropriate process [45] for a type of amplitude amplification procedure, thus enabling them to make decisions. Those facts also reflect that certain processes developed for quantum processing on artificial systems can naturally appear in biological systems. In any case, if similar process can appear in chemical or biological systems, it becomes clear that specialized non-local features can be more common or at least necessary to reach certain quantum cognition processes.

## 5. Final Processing Procedures and Analysis of Errors, Circuit Implementation, Decoherence and Account of Quantum Resources, and Applications

Grover’s quantum database search algorithm resulted in a dramatic reduction in the computational complexity of searching in an unsorted database [24]. Since then, many applications using this algorithm have emerged. The first quantum algorithm to approximate the mean was given by Grover [46] and certain other related algorithms have been proposed to estimate the mean and the median [47] based on amplitude estimation [48]. Following Grover’s seminal work, Durr and Hoyer [49] have proposed a quantum algorithm to determine the minimum of a function with a quadratic speed-up compared with the best possible classical algorithm. Another extension of Grover’s algorithm enables obtaining an approximate counting of the number of solutions for a search problem [50]. In the current section, we discuss several aspects related to the implementation presented in terms of possible extensions, application trends, and analysis of errors, circuit implementation notes, quantum decoherence limitations, and a comparison with alternative quantum and classical technologies.

### 5.1. Approach for a Generic Parallel Processing Model

All previous applications are reached through suitable final processing in from the amplitude amplification process. They allow realizing how certain global quantities are reached on data or information codified departing from an extended processing with several processors function together at the PP layer. In the approach presented before, the original single qubit state $|\varphi_{0}\rangle =\alpha_{0}{|0\rangle }_{0}+\alpha_{1}{|1\rangle }_{0}\equiv {\cos\theta |0\rangle }_{0}+e^{i\phi }\sin\theta {|1\rangle }_{0}$ contains information codified through θ and ϕ. Accordingly, each processing performed by each Bob${}_{j}$ develops a different specialized calculation, possibly integrating local additional information to reach and deliver larger composed states. Each one should perform only a local qubit operation $U_{j_{2j}}=e^{i\eta_{j}{\hat{n}}_{j}\cdot {\vec{\sigma }}_{2j}}$ (with ${\hat{n}}_{j}$ a three-dimensional vector, and ${\vec{\sigma }}_{2j}=\left(X_{2j},Y_{2j},Z_{2j}\right)$), delivering the output on each qubit labelled as 2j and containing the outcome codified. Each processing is synthetically characterized by ${\vec{n}}_{j}\equiv \eta_{j}{\hat{n}}_{j}$.

Even if each processing requires more space to be performed or a multi-output is required, certain ancilla qubits can be locally added to the process, particularly because of the additional local information inclusion. Thus, each Bob${}_{j}$ can first add quantum systems to each $|\varphi_{0}\rangle_{2j}$ as $|\varphi_{0}^{\prime }\rangle_{2j}\equiv {(\alpha_{0}|0\rangle }_{2j}+\alpha_{1}{|1\rangle }_{2j})⊗|\nu_{j}\rangle_{s_{j}}$ to then be processed into $|\varphi_{0}^{j}\rangle \equiv \alpha_{0}^{j}{|0\rangle }_{2j}⊗|\mu_{0,j}\rangle_{s_{j}}+\alpha_{1}^{j}{|1\rangle }_{2j}⊗{|\mu_{1,j}\rangle }_{s_{j}}$ (note the simplest case corresponds to $|\mu_{0,j}\rangle_{s_{j}}=|\mu_{1,j}\rangle_{s_{j}}\equiv {|\mu_{j}\rangle }_{s_{j}}$; however, the semi-local processing can consider the general expression). Each $|\nu_{j}\rangle_{s_{j}}$ and $|\mu_{k,j}\rangle_{s_{j}}$ with k=0,1;j=1,…,n are states of a higher dimension or states composed by several semi-local subsystems of each Bob${}_{j}$ (as considered in Figure 5). In this case, processing operations $U_{j_{2j},s_{j}}$ are not local, instead semi-local for the qubits belonging to each Bob${}_{j}$. Such additional qubits are labelled by a set of indices represented by $s_{j}$ (available to be used as ancilla qubits). In fact, such processes are reached by a set of unitary operators $u_{{a,b}_{s_{j}}}$ on the extended Hilbert space of each Bob${}_{j}$ in the following form:

(29) $$\begin{array}{ccc} U_{j_{2j},s_{j}} & = & \frac{1}{\sqrt{2}}\sum_{a,b=0}^{1}{|a\rangle }_{2j}\langle b|⊗u_{{a,b}_{s_{j}}} \end{array}$$

(30) $$\begin{array}{ccc} & & \mathrm{with}:\;u_{{0,0}_{s_{j}}}^{†}u_{{0,1}_{s_{j}}}+u_{{1,0}_{s_{j}}}^{†}u_{{1,1}_{s_{j}}}=0_{s_{j}} \end{array}$$

condition (30) is required to fulfill the unitary property on $U_{j_{2j},s_{j}}$. Thus, such operation takes the information of teleported state and also local information incoming from the environment of each Bob${}_{j}$. In addition, states $|\mu_{a,j}\rangle_{s_{j}}$ are:

(31) $$\begin{array}{c} \alpha_{a}^{j}|\mu_{a,j}\rangle_{s_{j}}=\frac{1}{\sqrt{2}}\left(\alpha_{0}u_{{a,0}_{s_{j}}}+\alpha_{1}u_{{a,1}_{s_{j}}}\right){|\nu_{j}\rangle }_{s_{j}},\;a=0,1 \end{array}$$

States $|\varphi_{0}^{j}\rangle$ are then transferred to Bob${}_{k}$ as in (16):

(32) $$\begin{array}{c} |\Psi_{k}\rangle =\sum_{i=1}^{n}\sqrt{p_{i}}{|i\rangle }_{C}⊗|\varphi_{0}^{i}\rangle \equiv \sum_{i=1}^{n}\sqrt{p_{i}}{|i\rangle }_{C}⊗{(\alpha_{0}^{i}|0\rangle }_{2k}⊗|\mu_{0,i}\rangle_{s_{k}}+\alpha_{1}^{i}{|1\rangle }_{2k}⊗|\mu_{1,i}\rangle_{s_{k}}) \end{array}$$

of course extending the C−SWAP operation on the semi-local systems $s_{j}$ of each Bob${}_{j}$ by moving them correspondingly into those $s_{k}$ of Bob${}_{k}$ through the change $SWAP_{2i,2k}\to SWAP_{2i,2k}⊗{⨂}_{\delta \in s_{j},\delta^{\prime }\in s_{k}}SWAP_{\delta ,\delta^{\prime }}$ in (15). Further analysis to select a subset of such superposition is identical to our previous development of the Grover-like algorithm because it is based on the orthogonal properties of the control basis ${\{|i\rangle }_{C}\}$. Thus, after the Grover-like process, the output state $|\Psi^{\left(R\right)}\rangle$ results into an approximation of the desired state $|A_{e}\rangle$. It can be expressed as follows:

(33) $$\begin{array}{ccc} |\Psi^{\left(R\right)}\rangle & = & \sum_{i=1}^{n}\alpha_{i}^{\left(R\right)}{|i\rangle }_{C}⊗{(\alpha_{0}^{i}|0\rangle }_{2k}⊗|\mu_{0,i}\rangle_{s_{k}}+\alpha_{1}^{i}{|1\rangle }_{2k}⊗|\mu_{1,i}\rangle_{s_{k}}) \end{array}$$

(34) $$\begin{array}{ccc} |A_{e}\rangle & = & \sum_{i\in K}\beta_{i}{|i\rangle }_{C}⊗{(\alpha_{0}^{i}|0\rangle }_{2k}⊗|\mu_{0,i}\rangle_{s_{k}}+\alpha_{1}^{i}{|1\rangle }_{2k}⊗|\mu_{1,i}\rangle_{s_{k}}) \end{array}$$

for a target subset K⊂{1,2,…,n}. After the last processing and transferring of information on a single party, a possible reduced post-measurement on the control system is necessary, particularly if the indexing needs to be changed, as considered in (17). This step could let to reach certain information coded in such state. It was tentatively suggested in Figure 5, $|\Psi^{\left(R\right)}\rangle$ contains the achievable state. It represents the ideal state $|A_{e}\rangle$ on which certain types of final processing and/or stochastic selection are ideally planned. Otherwise, an external state can be matched within the subset of outcomes. We will discuss certain aspects of both possibilities below.

### 5.2. Generic Applications for the Database Settlement with Partial Selection

Applications for the current procedure (first using parallel processing to set a database and subsequently performing a partial search) are mainly located across the reach of global information or otherwise for performing a query. Main search possibly needs be refined in a controlled manner for a concrete query according to specific problems. Thus, additional uses can include global information search or pattern matching on an already debugged database.

#### 5.2.1. Analysis of the Final Processing to Obtain Global Information from the Selected Database Subset

The discussion on global final processing has been presented in the previous sections, including several implementations related to the Grover algorithm. In fact, using an initial suitable codification on the initial state $|\varphi_{0}\rangle$ (basis encoding, amplitude encoding, or angle encoding), subsequently post-processing generates certain database with the procedure proposed maintaining the encoding inside each $|\varphi^{i}\rangle$. Accordingly, Grover-like procedure delivers a subset of such outcomes representing values to be averaged as a function of the type of encoding [47], optimized [49], and sorted [48,50]. Clearly, each one of those problems has been carefully treated to first introduce an effective encoding of information and subsequently a smart final processing $U_{f}$ to solve the global proposed task. Below, we bound the associated error transferred from the Grover-like algorithm of such a final processing, corresponding to the final layer of the procedure (FGP).

When the goal considers post-processing to reach certain global information from the selected outcomes in the database, then a final unitary global processing $U_{f}$ is expected on the entire $|A_{e}\rangle$ (or in fact on the approximated $|\Psi^{\left(R\right)}\rangle$ state). Such is the case for the aforementioned applications as the mean [46], median [47], amplitude estimation [48], and minimum finding [49] problems.

If no post-measurement is performed on the control, the errors can be estimated as follows:

(35) $$\begin{array}{ccc} D_{f} & = & \left|\right|U_{f}|A_{e}\rangle -U_{f}|\Psi^{\left(R\right)}{\rangle ||}_{K}=\left||\right|A_{e}\rangle -|\Psi^{\left(R\right)}{\rangle ||}_{K} \end{array}$$

(36) $$\begin{array}{ccc} F_{f} & = & |\langle A_{e}|U_{f}^{†}U_{f}|\Psi^{\left(R\right)}{\rangle |}^{2}={\left|\langle A_{e}|\Psi^{\left(R\right)}\rangle \right|}^{2} \end{array}$$

this implies the fidelity between both states. Note that the application of $U_{f}$ does not change the analysis of errors presented in the previous section. Indeed, both these quantities are related because:

(37) Df2=|||Ae〉−|Ψ(R)〉||K2=2(1−Re(〈Ae|Ψ(R)〉K))⟶2(1+Ff12)≥Df2≥2(1−Ff12)⟶Ff≥1−Df222

which, for the maximum outcome for D≈0.2929 (see Figure 7b), provides $F_{f}\geq 0.9160$.

#### 5.2.2. Quantum Pattern Matching with an External Quantum State

Quantum pattern matching is a widely known area related to quantum information, wherein techniques are developed to identify a quantum state codifying data inside a database. It is another alternate possibility for the final layer of the procedure (FGP). In the current stage of our development, a partial search has been already applied based on the quadratic speed-up in query complexity introduced by the Grover algorithm [44]. Such a subset contains the goal states where an additional external state is expected to be found. Thus, we need to find a concrete quantum state on an already limited superposition generated by the Grover algorithm. Note in certain cases the query can be performed from the beginning through a single post-selection. Here, we assume that the first reduction in database was already performed to subsequently determine a concrete state coinciding (or not) with the processing outcomes within the reduced database. Certain efficient procedures to determine a pattern included on a quantum state has been provided in [51]. Certain procedures are still based on Grover algorithm with the complexity of setting an oracle again and, in general, the entire settlement. When only few elements are present in the reduced selection, certain bit-parallelism techniques has been developed [52] and other statistical approaches has been attempted [53]. In this last trend, we show a short statistical method to discriminate matching.

Considering the ideal state (34) obtained through the post-selection using GAA, we assume that a certain external state $|\varphi^{*}\rangle$ (not necessarily known), with multiple identical copies available, should be identified as a member of the reduced subset of elements from the database: $\left\{|\varphi_{0}^{i}\rangle |i\in K\right\}$. Thus, we proceed to measure it on $|A_{e}\rangle$:

(38) $$\begin{array}{ccc} \frac{1}{\sqrt{P^{*}}}\langle \varphi^{*}|A_{e}\rangle & = & \frac{1}{\sqrt{P^{*}}}\sum_{i\in K}\beta_{i}\langle \varphi^{*}|\varphi_{0}^{i}\rangle {|i\rangle }_{C} \end{array}$$

(39) $$\begin{array}{ccc} P^{*} & = & |\langle \varphi^{*}|A_{e}\rangle {|}^{2}=\sum_{i\in K}|\beta_{i}{|}^{2}{\left|\langle \varphi^{*}|\varphi_{0}^{i}\rangle \right|}^{2} \end{array}$$

the control outcome state in case of success and the success probability, respectively. For simplicity, here we assume the most practical case with $\beta_{i}=\frac{1}{\sqrt{b}}$ and only $|\varphi_{0}^{i}\rangle =\alpha_{0}^{1}{|0\rangle }_{2k}+\alpha_{1}^{1}{|1\rangle }_{2k}$. The analysis of more complex cases including additional states $|\varphi_{0}^{i}\rangle =\alpha_{0}^{1}{|0\rangle }_{2k}⊗|\mu_{0,i}\rangle_{s_{k}}+\alpha_{1}^{1}{|1\rangle }_{2k}⊗{|\mu_{1,i}\rangle }_{s_{k}}$ is similar; however, it will require additional information regarding the nature of $|\mu_{j,i}\rangle_{s_{k}},j=0,1$, which is out of the scope of the current analysis. How can we identify if $|\varphi^{*}\rangle \in {\{|\varphi_{0}^{i}\rangle }_{2k}\left|i\in K\right\}$? In the current section, we present still-detectable differences in the statistics of such measurements if it belongs to the reduced subset provided by the Grover-like algorithm.

If $|\varphi^{*}\rangle \notin {\{|\varphi_{0}^{i}\rangle }_{2k}\left|i\in K\right\}$ and as instance, $\left\{\theta_{i},\phi_{i}|i\in K\right\}$ are uniformly distributed on the Bloch sphere, and it can be reasonably assumed that $\frac{1}{\sqrt{P^{*}}}\langle \varphi^{*}|A_{e}\rangle$ is closer in average from $|{\hat{\phi }}_{C}\rangle \equiv \sum_{i\in K}\frac{1}{\sqrt{b}}{|i\rangle }_{C}$. Accordingly, if we are able to measure such a state, there is a large probability for the first affirmation (note for the case $n=2^{p},p\in Z$ this operation can be performed using a series of Hadamard gates). Thus, if such an outcome is obtained on the control, the last success probability is:

(40) $$\begin{array}{ccc} P_{C} & = & \frac{1}{P^{*}}|\langle {\hat{\phi }}_{C}|⊗\langle \varphi^{*}|A_{e}\rangle {|}^{2}=\frac{1}{b^{2}P^{*}}{\left|\sum_{i\in K}\langle \varphi^{*}|\varphi_{0}^{i}\rangle \right|}^{2}=\frac{|\sum_{i\in K}\langle \varphi^{*}|\varphi_{0}^{i}\rangle {|}^{2}}{b\sum_{i\in K}{\left|\langle \varphi^{*}|\varphi_{0}^{i}\rangle \right|}^{2}} \end{array}$$

Otherwise, measuring $|\varphi^{*}\rangle$ does not necessarily imply that it is one of the $|\varphi_{0}^{i}\rangle$ because those states are not necessarily orthogonal. Nonetheless, it is expected that only one of $\langle \varphi^{*}|\varphi_{0}^{i}\rangle =1$. Here, we assume that all elements of the database are different. In fact, if $|\varphi^{*}\rangle$ can be repeatedly measured, larger possibilities can be identified. Depending on the database, there are extensive possibilities pertaining to the probability distribution of $\left\{|\varphi_{0}^{i}\rangle |i\in K\right\}$. Expressing $|\varphi_{0}^{i}\rangle =\cos\frac{\theta_{i}}{2}|0\rangle +e^{i\phi_{i}}\sin\frac{\theta_{i}}{2}|1\rangle ,i\in K$ and $|\varphi^{*}\rangle =\cos\frac{\theta^{*}}{2}|0\rangle +e^{i\phi^{*}}\sin\frac{\theta^{*}}{2}|1\rangle$, $\langle \varphi^{*}|\varphi_{0}^{i}\rangle =\cos\frac{\theta^{*}}{2}\cos\frac{\theta_{i}}{2}+e^{i(\phi_{i}-\phi^{*})}\sin\frac{\theta^{*}}{2}\sin\frac{\theta_{i}}{2}$. Considering that $\left\{\theta_{i},\phi_{i}|i\in K\right\}$ is uniformly distributed on the Bloch sphere and using the Haar measure [54], certain expected values associated with $\langle \varphi^{*}|\varphi_{0}^{i}\rangle$ are shown by direct calculation. They are developed in Appendix C. The outcomes are fundamental for obtaining $P^{*},P_{C}$.

**Figure 10:**
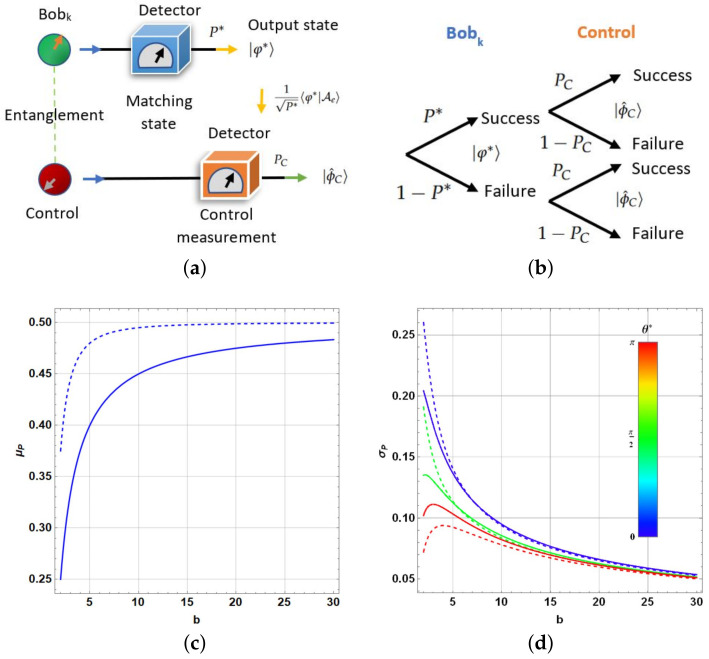
(**a**) Measurement process for pattern matching represented by the external state $|\varphi^{*}\rangle$. (**b**) Tree diagram for the measurement process matching the pattern $|\varphi^{*}\rangle$ on Bob${}_{k}$ and subsequently identifying an improbable control state $|{\hat{\phi }}_{C}\rangle$. The figure of interest is $P\equiv P^{*}\left(1-P_{C}\right)$. (**c**) Mean of *P*, and (**d**) Standard deviation of *P*, as function of *b* and $\theta^{*}$. In the last two cases, dashed lines correspond to one exact matching in the database subset.

The previous measurement procedure on Bob${}_{k}$ and control is illustrated in Figure 10a. Accordingly, we can consider the four total probabilities as instance in the process (otherwise conditional ones): $P^{*}P_{C},P^{*}\left(1-P_{C}\right),\left(1-P^{*}\right)P_{C}$, or $\left(1-P^{*}\right)\left(1-P_{C}\right)$ (see Figure 10b). By analyzing each possibility, $P\equiv P^{*}\left(1-P_{C}\right)$ is found to exhibit a distribution with reasonable detectable differences between the cases when $|\varphi^{*}\rangle$ belongs to the subset of the database obtained by Grover algorithm.

Furthermore, considering the outcomes (A20)–(A23), obtaining the expressions for the expected value of *P*, $\mu_{P}$ (A26) and their variance $\sigma_{P}^{2}$ (A27) is feasible, see the development in Appendix C. Note that $\sigma_{P}^{2}$ depends on $\theta^{*}$. Figure 10c and d show such quantities (dashed lines for the case of a perfect matching inside the database). Figure 10c shows a finite gap in $\mu_{P}$ when $|\varphi^{*}\rangle$ belongs to the database-reduced subset. This gap changes from $\frac{1}{8}$ to zero when *b* is increased, rendering detectability in principle to each situation for low values of *b*. If several copies are available, the procedure can be repeated to detect the belonging by analyzing the emerging statistics. An efficient process of multiple teleportation and Grover amplitude amplification is required to reproduce the process. Despite repetitions, if $|\varphi^{*}\rangle$ is unknown, determining if such a pattern in the form of $|\varphi^{*}\rangle$ is in the reduced subset is useful. Differences in $\sigma_{P}$ are lower and in addition they depend on $\theta^{*}$. It reduces the possibility to detect differences based on this last statistical quantity.

Finally, we still need to bound the outcome for $\mu_{P}$ by considering the real outcome state incoming from the Grover-like procedure $|\Psi^{\left(R\right)}\rangle$ and the real expected value $\mu_{P}^{\prime }$. Using the Cauchy–Schwarz inequality and the properties of the inner product:

(41) $$\begin{array}{ccc} \Delta \mu_{P} & = & |\mu_{P}-\mu_{P}^{\prime }|\\ & = & \left|\bar{\left(|\langle \varphi^{*}|A_{e}\rangle {|}^{2}-|\langle {\hat{\phi }}_{C}{\left|⊗\langle \varphi^{*}|A_{e}\rangle \right|}^{2}\right)}-\bar{\left(|\langle \varphi^{*}|\Psi^{\left(R\right)}\rangle {|}^{2}-|\langle {\hat{\phi }}_{C}{\left|⊗\langle \varphi^{*}|\Psi^{\left(R\right)}\rangle \right|}^{2}\right)}\right|\\ & = & \left|\bar{\mathrm{Tr}\left(|\varphi^{*}\rangle \langle \varphi^{*}|⊗(1-|{\hat{\phi }}_{C}\rangle \langle {\hat{\phi }}_{C}|)(|A_{e}\rangle \langle A_{e}\left|-\right|\Psi^{\left(R\right)}\rangle \langle \Psi^{\left(R\right)}|)\right)}\right|\\ & \leq & |\mathrm{Tr}(|\varphi^{*}\rangle \langle \varphi^{*}|)||\mathrm{Tr}(1-|{\hat{\phi }}_{C}\rangle \langle {\hat{\phi }}_{C}|)\left|\right|\bar{\mathrm{Tr}(|A_{e}\rangle \langle A_{e}\left|-\right|\Psi^{\left(R\right)}\rangle \langle \Psi^{\left(R\right)}|)}|\\ & = & \left(b-1\right)|\bar{\mathrm{Tr}((|A_{e}\rangle -|\Psi^{\left(R\right)}\rangle )(\langle A_{e}|+\langle \Psi^{\left(R\right)}|)}|\\ & = & \left(b-1\right)|\bar{(\langle A_{e}|+\langle \Psi^{\left(R\right)}|)\cdot (|A_{e}\rangle -|\Psi^{\left(R\right)}\rangle )}|\leq 2\left(b-1\right)D \end{array}$$

observing that such an error increases with the function of *b*, as expected. Nonetheless, for low values of *b* as those in Figure 9, it is still sufficiently bounded and $\mu_{P}^{\prime }\approx \mu_{P}$.

### 5.3. Grover Circuit Implementation for the Current Approach

Several proposals implementing the Grover algorithm as a quantum circuit have been reported through seminal works. There are differences between the cases where either Grover’s algorithm is used to determine a unique solution or multiple ones. In the first case, for instance, Grover’s circuit has been depicted in [55,56,57], with the typical value for the steps $O(\sqrt{1/n})$. In [58], a circuit implementation of a key search for the Advanced Encryption Standard (AES) is analyzed; however, the capability of modifying Grover’s algorithm is mentioned; therefore, it can cope with a larger but still unknown number of solutions. Nevertheless, [59] presents a variant of the algorithm capable of acquiring a solution even when the number of solutions is greater than one. In the same trend, in [60], the design of a circuit for an extended Grover’s algorithm for any dimension is presented using a decomposed *n*-qudit Toffoli gate, thus considering the concept of qudits. For the current approach, the circuit presented in [61] is useful. It is the most general form for algorithm construction due to the way they present the oracle and Grover diffusion operations. Nevertheless, as proved feasible in previous studies, the circuit can be implemented in a larger environment with more than one solution, guided by the ideas presented in [62], where states are constructed as a combination of several qubits. In our case, it is particularly appropriate for the design of the control state.

In the current procedure, the operators $U_{e}$ and $U_{m}$ should be implemented as the states $|A_{e}\rangle =\sum_{k\in K}\beta_{k}|A_{k}\rangle$ and $|\Psi^{\left(0\right)}\rangle =\frac{1}{\sqrt{n}}\sum_{i=1}^{n}|A_{i}\rangle$, respectively. Although the implementation is possible in principle, the knowledge of the $\beta_{k}$ coefficients in the target state $|A_{e}\rangle$ becomes difficult to acquire, rendering the implementation impractical on a generic quantum circuit. Otherwise, as analyzed in the previous section, an evenly distributed target state becomes better than another arbitrary one (despite exhibiting a suitable performance for lower searches b≪n. Thus, in such a case, it becomes more practical to consider $\beta_{k}=\frac{1}{\sqrt{b}}$ than to include local post-processing on the control to modify the coefficients, if necessary. Coefficients $\sqrt{p_{i}}$ on the control can be implemented based on the design; however, if the target state is selected with $\beta_{k}=\frac{1}{\sqrt{b}}$, such initial design will become unproductive. Thus, the election $\sqrt{p_{i}}=\frac{1}{\sqrt{n}}$ becomes the most convenient.

For the last election, the quantum circuits introduced in the literature for Grover algorithm are still adequate with certain modifications for the current development by operating them on the control states ${|i\rangle }_{C}$ as the elective states (because they are maximally entangled with the processed outputs $|\varphi_{0}^{i}\rangle$ or $\alpha_{0}^{i}{|0\rangle }_{2k}⊗|\mu_{0,i}\rangle_{s_{k}}+\alpha_{1}^{i}{|1\rangle }_{2k}⊗{|\mu_{1,i}\rangle }_{s_{k}}$). The control state can be a genuine quantum state of dimension *n* or a multipartite state of qubits with dimension $n=2^{p}$ expressed in the binary notation ${|i\rangle }_{C}={⨂}_{v=1}^{p}|i_{v}\rangle$ with an appropriate length *p* and $i=\sum_{v=1}^{p}i_{v}2^{i-1},i_{v}\in \left\{0,1\right\}$. In the last case, applying the controlled operation C−U as developed in Appendix A is appropriate for this structure.

**Figure 11:**
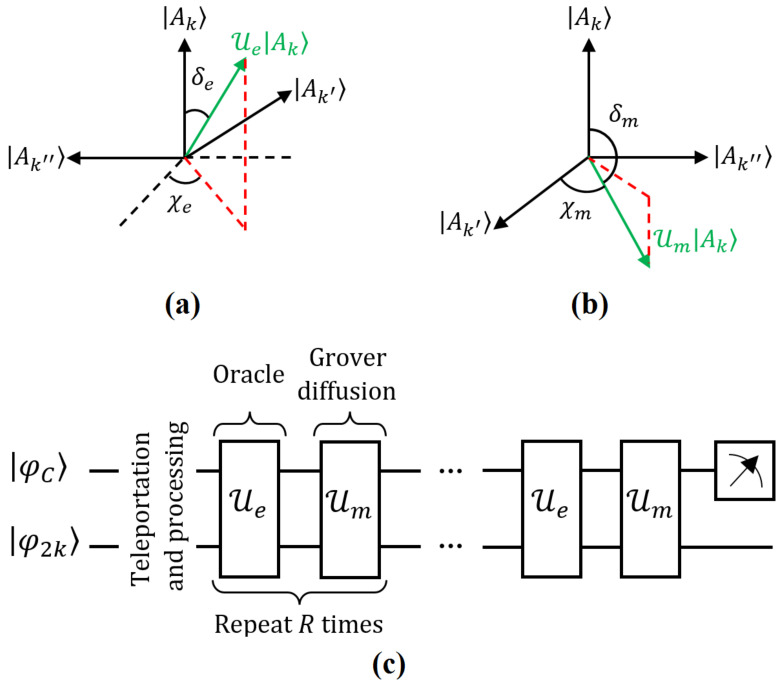
(**a**) Effect of $U_{e}$ on each $|A_{k}\rangle ,k\in K$ as a multidimensional rotation by angles $\delta_{e}$ and $\chi_{e}$ with respect to itself and other $|A_{k}^{\prime }\rangle ,k^{\prime }\in K$. (**b**) Effect of $U_{m}$ on each $|A_{k}\rangle$ as a multidimensional rotation by angles $\delta_{m}$ and $\chi_{m}$ with respect to itself and other $|A_{k}^{\prime }\rangle$. (**c**) Simplified Grover circuit considering the oracle $U_{e}$ and the Grover diffusion operator $U_{m}$, both repeated *R* times after teleportation and processing, with a possible control state measurement at the end.

From (22), operator $U_{e}$ for the multiple post-selection presented in Section 4.2.2 is an oracle exhibiting the following form:

(42) $$\begin{array}{c} U_{e}|A_{k}\rangle =\left\{\begin{array}{cc} |A_{k}\rangle -\frac{2}{b}\sum_{k^{\prime }\in K}|A_{k^{\prime }}\rangle , & k\in K\\ |A_{k}\rangle , & k\notin K \end{array}\right. \end{array}$$

such operation represents a rotation on the subspace $\mathrm{span}(\{|A_{k}\rangle |k\in K\})$. It tilts $|A_{k}\rangle$ by an angle $\delta_{e}=\arcsin\left(1-\frac{2}{b}\right)$ with respect to itself and an angle $\chi_{e}=\arccos\left(\frac{1}{\sqrt{b-1}}\right)$ with respect to the other vectors in the subspace (if b≠1, see Figure 11a). Thus, such rotation does not depend on the structure of $|A_{e}\rangle$ but only on *b* and the selection of *K*.

Similarly, the operator $U_{m}$ for the reflection respect to $|\Psi^{\left(0\right)}\rangle$ has the following effect:

(43) $$\begin{array}{c} U_{m}|A_{k}\rangle =-(|A_{k}\rangle -\frac{2}{n}\sum_{j=1}^{n}|A_{j}\rangle ) \end{array}$$

a tilt of $|A_{k}\rangle$ by an angle $\delta_{m}=\frac{\pi }{2}+\arcsin\left(1-\frac{2}{n}\right)$ respect itself and an angle $\chi_{m}=\arccos\left(\frac{1}{\sqrt{n-1}}\right)$ respect the other vectors in the entire space for the control state (see Figure 11b). Again, such a rotation only depends on *n*. A schematic representation of the amplitude amplification is shown in Figure 11c (ancilla qubits are not illustrated).

If each control state ${|i\rangle }_{C}$ is performed by a group of *p* qubits ($n=2^{p}$) as depicted previously: $|\varphi_{C}\rangle =\sum_{s=0}^{2^{p}-1}\sqrt{p_{s}}{|s\rangle }_{C}$ (${|s\rangle }_{C}$, briefly expressed in base-10 notation). Accordingly, last operations can be generated by entangling operations between them and the states for qubit 2k of Bob${}_{k}$; nonetheless, it involves few qubits at time. In particular, in such a scheme, $U_{m}=H^{⊗p}\cdot (2|0\rangle \langle 0{|}^{⊗p}-1^{⊗p})\cdot H^{⊗p}$ [63]. Another approach has been introduced in [62] in terms of single operations. In any case, the state $|\Psi^{\left(R\right)}\rangle \approx |A_{e}\rangle$, with $\beta_{k}=\frac{1}{\sqrt{b}}$, is obtained.

### 5.4. Limitations Imposed by Possible Decoherence Effects

The entire process undergoes multiple teleportation and parallel post-processing; nonetheless, the final treatment for gathering the multiple outcomes has been performed assuming well-functioning gates, which, in fact, are finally reduced to physical interactions between imperfect systems. Quantum decoherence is introduced by the environment that affects the setups settled either on photonic systems or on matter systems. Although photonic implementations are better recommended, quantum information involved in the process should be stored, particularly the information related to the database. Although decoherence effects are mild for photonic implementations, it is not true for based-matter systems. Such undesirable effects on gate are hardly analyzed as quantum open systems using Linblad or Redfield equations [64]; otherwise approximations are performed using non-Hermitian Hamiltonians [65].

However, both approaches become exceedingly complex when many gates are introduced in a complex procedure. In another trend, this problem has already been tackled for the most common gates implemented in quantum processing on matter through certain practical considerations [66]. Certain gates as NOT, Hadamard, $C^{a}NOT_{b}$, Toffoli, etc., (all of which are involved in the current procedure) have been analyzed using the Lindblad equation approach for Nuclear Magnetic Resonance (NMR). As a result, guidelines for quantum circuit designers were reported for their fidelity behavior due to decoherence [67] under amplitude and phase damping as the most representative examples of noise. As a result, certain considerations were stated: (a) deeper circuits of course exhibit lower fidelity (processing more qubits), (b) multiqubit gates do not necessarily exhibit lower fidelity than single gates, and (c) shorter timescales to reach each gate still maintain fidelities close to one.

In the Noisy Intermediate Scale Quantum (NISQ) technologies, current global coherence times are in the range of 50–100 μs, thus dealing with fidelities routinely implemented above 0.99 for single qubits gates. This is also true for certain two-qubit gates. In the current technologies, individual operation times are in the order of nanoseconds; thus, large circuits can still be addressed during the coherent time-window [68]. This implies that circuits with gates in the order of tens can be implemented. Table 2 reports the gates for the initial part of the process, their type by process (MT and PP as before), and the number of qubits involved. More than a half are single-qubit gates. Thus, implementation on matter-based technologies is only in the technological frontier.

If controlled operations are necessarily involved, the depth of the process increases requiring deeper circuits involving 4−qubits operations in the current technological limit [69]. Grover implementation based on initial and final evenly distributed states (as required in the current development) demands the same resources as the traditional Grover algorithm because the outcomes of the database are previously allocated on the same party. Thus, technological developments in this direction cover the current analysis. As [43] has stated, few Grover implementations and concrete applications have been provided, mainly due to difficulties to state the oracle in the procedure [70,71].

In the classical domain, parallel processing is understood as a computing process, dividing a large task into many smaller ones. They are executed on several computation nodes, resulting in swifter completion. This requires certain large tasks to be able to be executed simultaneously, nonetheless preserving the sequencing of their constituents. Although each processor can use its own memory, computation nodes also require access to data, software, or peripheral devices to obtain a concrete global outcome or process by sharing a single physical database and memory [72]. In fact, an integrated processing on such a database requires access to distributed locations where outcomes or data are stored but being read, load, and processed by a single memory to obtain the final outcome: a global processing or query, aspects superseded in principle by a quantum approach [73].

By comparing the current process with other proposals of quantum database queries, most of them assume that the database is already settled [74,75] without analyzing the complexity. Instead, a recent current development successfully addresses the entire procedure [43] employing AAE [35], a method based on a variational approach, which loads the sufficient approximation to the objective database. In our approach, we are using exact non-local procedures, which require dominion of challenged techniques controlling non-local resources, thus requiring a faithful construction and distribution at the edge of the current quantum technologies.

### 5.5. Image Matching on a Large Dataset: An Example

To further clarify the procedure presented, we discuss an example regarding the analysis of a large set of images to analyze their invariant properties under certain transformations. Consider a set of images of m×m pixels. Such a simple set can have $2^{m^{2}}=65,536$ different images in total. It becomes harder when it is scaled (m≫4). Suppose that an unknown subset of $n\leq 2^{m}$ (not repeated) is being considered. They are locally stored in different parts of a quantum memory, where in addition to the first qubit from a Bell pair is present in each semi-local set (another one is in possession of Alice to perform the teleportation to those places). Each local image $|\nu_{j}\rangle_{s_{j}}={⨂}_{k=1}^{m}|{\nu_{j}}_{k}^{4}\rangle$ is represented based on their pixels ${\nu_{j}}_{k}^{4}\in \left\{0,1\right\}$ in base-2, being 0 white and 1 black, whereas $\nu_{j}\in \left\{0,1,\ldots ,2^{m}-1\right\}$ is expressed in base-10. Note that, in this case, states storing the images and their different translations are orthogonal (except with invariant images under those translations). In terms of the previous development presented, $|\mu_{a,i_{s_{j}}}\rangle_{s_{k}}$, with i,a∈{0,1} being the images obtained after each rotation departing from the image *i*: horizontal a=0 and vertical a=1.

**Figure 12:**
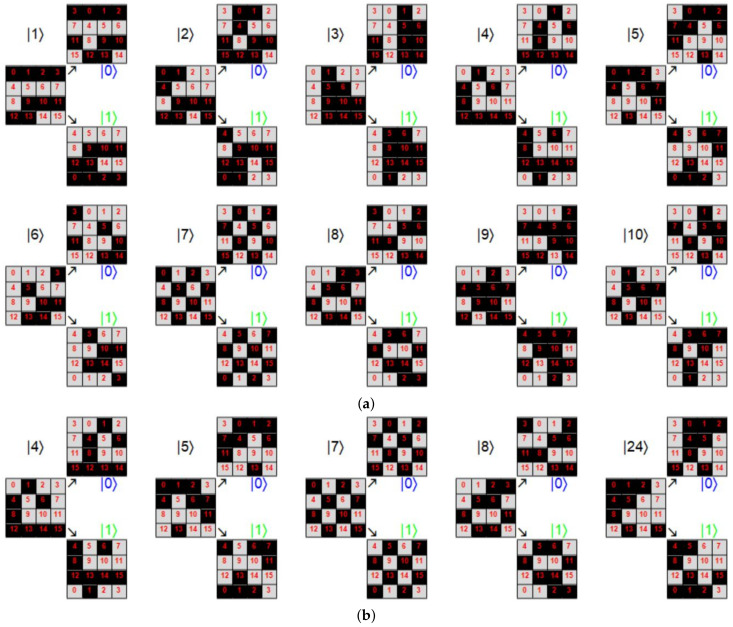
Pattern matching problem with translations. (**a**) Ten first images of an original dataset with the image on the left stored as local information, and those on the right obtained as an outcome by cyclic right-forward (|0〉) or upward (|1〉) translations in superposition obtained under parallel processing. (**b**) First five images in the subset of the previous dataset obtained by amplitude amplification on the cases with exactly 8 dark pixels.

An original qubit in possession of Alice $|\varphi_{0}\rangle_{0}=\alpha_{0}{|0\rangle }_{0}+\alpha_{1}{|1\rangle }_{0}$ will decide the cyclic right-forward (|0〉) or upward (|1〉) translations (${u_{0,0}}_{s_{j}}$ and ${u_{1,1}}_{s_{j}}$, respectively, not developed here) on each image (and their superposition weights). If more complex qubits are teleported, a more diverse set of operations can be simultaneously considered. A control system identical to (1) manages the multiple teleportation. Figure 12a illustrates the case m=4 and the first 10 elements in a distributed dataset. Each pixel is originally numbered from 0 to 15 to fulfill the notation previously established in $|\nu_{j}\rangle_{s_{j}}$. Thus, the state indexed by ${|1\rangle }_{C}$ is $|\nu_{1}\rangle_{s_{1}}=|1111000001111100\rangle$. After the translations, the numbering has been preserved as a reference; however, the original numeration for the qubits storing each pixel is maintained. Thus, translation is a set of swapping between the states of the qubits storing each image. Thus, as instance: $|\mu_{0,1}\rangle_{s_{k}}=|1111000010110110\rangle$ and $|\mu_{1,1}\rangle_{s_{k}}=|0000011111001111\rangle$. Applying the controlled operation:

(44) $$\begin{array}{c} {U_{j}}_{2j,s_{j}}={|0\rangle }_{2j}\langle 0|{u_{0,0}}_{s_{j}}+{|1\rangle }_{2j}\langle 1|{u_{1,1}}_{s_{j}} \end{array}$$

which clearly fulfills (30), we finally obtain the distributed database of outcoming images depicted by $|\Psi_{k}\rangle$ in (16).

Accordingly, several problems can be derived from here. We assume that the image does not belong to the original set, but probably to the translated set of outcomes, thus rendering parallel processing necessary. Furthermore, if a single image matching is directly performed on a certain index or still if the search is performed on a set of indexes, the use of amplitude amplification can be performed for comparison stochastically (by instance using weak measurements as $W_{0},W_{1}$) or with the procedure presented in Section 5.2.2 with the external image being searched. The final stochastic search functions because of the orthogonality of the state images. If it is successful, the remaining state in the control system and in $|\varphi_{0}^{i}\rangle_{2k}$ will store the index location(s) and the translation operation(s) performed.

Instead, if we treat only a selected subgroup to reach a certain global property, we can use the Grover-like amplitude amplification method first, based on the knowledge of the indexes fulfilling the search criteria. For instance, if only the images with exactly 8 dark pixels are considered (see Figure 12b), then Grover algorithm will select such subset in $|\Psi^{\left(R\right)}\rangle$, ideally in $|A_{e}\rangle$. In the last expression, in agreement with our convergence analysis, the most precise solution in the Grover procedure is achieved when $p_{i}=\frac{1}{N}$ and $\beta_{i}^{2}=\frac{1}{b}$. We assume such prescriptions as follows: additionally, we consider $\alpha_{0}=\alpha_{1}=\frac{1}{\sqrt{2}}$. In this case, measuring the qubit 2k on the basis |±〉, $\langle \pm |A_{e}\rangle$ normalized becomes:

(45) $$\begin{array}{ccc} {\langle \pm |A_{e}\rangle }_{norm} & = & {\left(\frac{1}{\sqrt{2b}}\sum_{i\in K}{|i\rangle }_{C}⊗{(|\mu_{0,i}\rangle }_{s_{k}}\pm |\mu_{1,i}\rangle_{s_{k}})\right)}_{norm}\\ & = & \frac{1}{\sqrt{N}}\left(\Delta^{\pm }\sum_{i\in K_{I}}{|i\rangle }_{C}⊗|\mu_{i}^{I}\rangle_{s_{k}}+\sum_{i\in {\tilde{K}}_{I}}{|i\rangle }_{C}⊗{(|\mu_{0,i}\rangle }_{s_{k}}\pm |\mu_{1,i}\rangle_{s_{k}})\right) \end{array}$$

where $K_{I},{\tilde{K}}_{I}$ are the subsets of invariant and non-invariant figures (as ${|7\rangle }_{C}$ in Figure 12a,b). Additionally, $\Delta^{\pm }=1\pm 1$ and N±=Δ±2NI+2∑i∈K˜I(1±Re〈μ0,i|μ1,i〉), being $N_{I}$ the number of equivalent images under both translations. Using post-measurement post-processing, when the measurement outcome is |−〉, the state in (45) will contain only the non-invariant images, with a number of terms $\left(b-N_{I}\right)\geq \frac{N^{-}}{4}$, thus containing global information regarding the global set of images in terms of their invariance. Such state also contains a set of entangled states $∼{(|\mu_{0,i}\rangle }_{s_{k}}-|\mu_{1,i}\rangle_{s_{k}})$ regarding the similitude among the images upon both transformations. In both cases, certain global information characterizing the entire set has been obtained. Nevertheless, further information should be extracted with additional algorithm involving global processing depending on specific problems.

## 6. Conclusions

In the current report, we have developed a tentative procedure to set a database using multiple teleportation for further parallel processing, two tasks commonly intertwined in the classical computing domain, where reading and writing on local processors are performed, and their number limits the scalability. Quantum information systems circumvent such limitations because dense coding is possible owing to state superposition. In our developed procedure, we have used a basic unit of information in the form of a qubit; however, it can be easily extended considering a larger quantum system, particularly those composed of two-level quantum systems [22]. Such resources are multiply teleported in the current strategy to set a copy in superposition, to then be processed in parallel under differentiated computing procedures working as local processors. Such a procedure possibly introduces additional local information. This combination of steps allows setting a non-local distributed database. It can be moved via entanglement on a single party stating a final indexed local database.

After a partial search based on amplitude amplification has been performed to select certain outcomes on demand, a subset of outcomes is set (either through the index stated by the control state or otherwise introduced in the oracle, which could be constructed using similar repeated external resources). Such a search has been featured in terms of their efficiency as a function of the parameter b/n, the fractional size of the database selection. In addition, we have analyzed their convergence in certain cases of interest, determining as the best scenario an initial state encompassing the entire outcomes with the same amplitude evolving on another reduced state with also balanced amplitudes for further tasks, only containing certain desired outcomes.

A tentative ending step considers either a final global processing to reach certain global information obtained from the reduced database or a pattern matching with certain possible external state. In the current literature of quantum parallel processing and amplitude amplification exists exemplary processes, thus averaging, optimizing, and sorting the information. For pattern matching, we have analyzed an alternative procedure (to the direct amplitude amplification) to identify if such a pattern is present or not within the reduced database. It is based on the analysis of certain statistical quantities unveiling differences: an involved probability *P* in the process, which experimentally unveils the pattern matching. Nonetheless, such a procedure requires the entire process repetition. Otherwise, when it is possible, the amplitude amplification procedure can be performed since the beginning with the involvement in the oracle of the concrete external state to be matched.

If the distance among processing parties is approximately a few nanometers or thousands of kilometers as a part of a distributed computer design, in our development, the dominion of teleportation and particularly of the non-local activation to transfer and control quantum information among several parties still require a notable advancement of quantum technologies. Nonetheless, we have noticed based on the development that operations involved in the current proposal naturally appear to occur in the nanoscale as part of the interactions among molecules or still more complex structures in the biochemical domain. An obvious question arises here: have those structures evolved more complex forms as brains, as shown by a type of quantum cognition enabling them to make decisions? Other questions appear regarding the self-measurement capacity of those structures in the making-decisions process, reproducing certain notable experiments as the Dirac 3-polarizer experiment or other similar experiments involving the Zeno effect, whose continuous measuring effects mimic certain decisions-making aspects in the brain, strengthening the possibility of self-measurement there.

Thus, we have also discussed how certain controlled processing and information transferring operations can be performed through non-local multi-processing by using entanglement and controlled measurements, both in the current frontier of quantum technologies. Non-local features presented here enable stating an alternative approach (despite the limit of our current quantum control and technologies) to set parallel processing on local memories where a database of information have been suitably settled. If chemical or biological systems are able to set on them similar non-local processing to evolve, an interesting question is already being considered in the current disruptive literature pertaining to quantum machine learning, quantum artificial intelligence, and quantum cognition. Dominion of such operations becomes relevant because they illustrate how to perform controlled operations at a distance via entanglement. Such type of control is feasible in the future of quantum processing and communications. Thus, the quantum world must still develop superior procedures over our classical world approaches and current technological capacities.

## Figures and Tables

**Table 1:** Key states, operators, and main quantities through each step (MT, PP, GAA, and FGP) of the procedure.

Symbol	Step	Description
$|\psi_{0}\rangle$	MT	Original qubit state to be teleported
$|\psi_{C}\rangle$	MT	Control state to manage the final receiver in multiple teleportation
$p_{i}$	MT	Superposition probabilities for each receiver in multiple teleportation
$|\beta_{ij}\rangle$	MT	Entangled resources for teleportation in the form of Bell states
$C^{a}NOT_{b},H_{0}$	MT	Controlled NOT and Hadamard gates to manage the multiple teleportation
$|\Psi_{0}\rangle$	MT	Initial state during the multiple teleportation process
$|\Psi_{\mathrm{pm}}\rangle ,|\Psi_{teleported}\rangle$	MT	Post-measurement and telported state at the end of multiple teleportation process
$U_{i_{k}}$	PP	Local processing operators on the qubit *k* in possession of party *i*
$C_{U}$	PP	Controlled operation to apply local processing $U_{i_{k}}$ on each receiver
$|\varphi_{0}^{i}\rangle$	PP	Output state from each local processing
$|\Psi_{\mathrm{pp}}\rangle$	PP	Global post-processing output state
C−SWAP	PP	Controlled SWAP operations to transfer the output states on a single party
$|\Psi_{k}\rangle$	PP	Post-processing output state transferred on a single party
$\gamma_{i}^{l}$	PP	Transformation coefficients of a tentative basis measurement on the control state
$P_{c_{l}}$	PP	Success probability for each outcome of the control state measurement
$U_{e},U_{m}$	GAA	Oracle and Grover diffusion operators
$|A_{e}\rangle$	GAA	Target state for the amplitude amplification process
$|\Psi^{\left(s\right)}\rangle$	GAA	Output state in the step *s* of the amplitude amplification
$\alpha_{j}^{\left(s\right)},\beta_{j}$	GAA	*s*-step output state and target coefficients
D,F	GAA	Cartesian distance norm and fidelity between the Grover output and ideal states
*R*	GAA	Necessary number of repetitions of Grover-like procedure to obtain the closest outcome
$U_{f}$	FGP	Generic final global processing operator

**Table 2:** Depth and number of each type of gate involved through the different steps of the process (DT and PP) without considering operations among faraway parties. Instead, PP* considers the operations among faraway parties.

Depth:	1			2		3	4	Total
Gates:	X	Z	*H*	${\mathrm{SWAP}}_{\mathrm{ab}}$	$C^{a}U_{b}$	Toffoli	Controlled	
MT	*n*	*n*	1	0	0	*n*	0	3n+1
PP	0	0	0	n−1	*n*	1	0	2n
PP*	2(n−1)	n−1	0	2n−1	5n−4	n−1	*n*	12n−9

## Data Availability

Not applicable.

## Abbreviations

The following abbreviations are used in this manuscript:

AAE	Approximate Amplitude Encoding
AES	Advanced Encryption Standard
DBMS	Database management system
GAA	Grover’s Amplitude Amplification
GFP	Global Final Processing
MT	Multiple teleportation
NISQ	Noisy Intermediate Scale Quantum
PM	Pattern Matching
PP	Post-processing
QIMP	Quantum Image Processing
QKD	Quantum Key Distribution
QPM	Quantum Pattern Matching

## Appendix A. Control on Faraway Non-Local Resources

Controlled operations on faraway non-local parties, as that C−U in (13), can be achieved as:

(A1) $$\begin{array}{ccc} C-U & = & \prod_{i=1}^{n}C_{i}^{C}U_{i_{2i}}\\ & & \mathrm{where}:\;C_{i}^{C}U_{i_{2i}}\equiv \sum_{i\neq j=1}^{n}{|j\rangle }_{C}\langle j|⊗1_{2j}+{|i\rangle }_{C}\langle i|⊗U_{i_{2i}} \end{array}$$

nonetheless, each factor cannot be performed directly (assuming that the control and qubit 2i cannot be moved from their far locations). Nevertheless, they can be achieved via LOCC with the support of an entangled state ${|T\rangle }_{ab}$ where qubit *a* is in possession of Alice/Control systems and *b* is sent to Bob${}_{i}$:

(A2) $$\begin{array}{ccc} |\psi \rangle & = & \sum_{j=1}^{n}\sqrt{p_{j}}{|j\rangle }_{C}|\phi_{j}\rangle_{\left\{2i\right\}}|\psi_{j}\rangle_{2i}⊗{|T\rangle }_{ab}\\ & & {\mathrm{with}:\;|T\rangle }_{ab}=\frac{1}{\sqrt{n}}\sum_{k=1}^{n}{|kk\rangle }_{ab} \end{array}$$

which represents a general state as in our development, with the qubit 2i explicitly separated from the remaining system of even subscripts represented by the state with subscript {2i}. A direct calculation shows that a such state can be written as:

(A3) $$\begin{array}{ccc} |\psi \rangle & = & \sum_{j,k=1}^{n}\sqrt{\frac{p_{j}}{n}}{|jk\rangle }_{Ca}⊗|\phi_{j}\rangle_{\left\{2i\right\}}|\psi_{j}\rangle_{2i}{|k\rangle }_{b} \end{array}$$

considering a basis change defined as:

(A4) $$\begin{array}{c} {|jk\rangle }_{Ca}=\sum_{u,v=1}^{n}T_{jk,uv}{|B_{uv}\rangle }_{Ca} \end{array}$$

where $|B_{uv}\rangle_{Ca}$ are possibly entangled states in general. Thus:

(A5) $$\begin{array}{ccc} |\psi \rangle & = & \frac{1}{\sqrt{n}}\sum_{u,v=1}^{n}|B_{uv}\rangle_{Ca}⊗\sum_{j,k=1}^{n}\sqrt{p_{j}}T_{jk,uv}|\phi_{j}\rangle_{\left\{2i\right\}}|\psi_{j}\rangle_{2i}{|k\rangle }_{b} \end{array}$$

The nature of the $T_{jk,uv}$ coefficients selects all of them with the absolute value of $\frac{1}{\sqrt{n}}$ in the following way: for each u,v pair, then $T_{jk,uv}\neq 0$ only for *n* pairs from the $n^{2}$ combinations of j,k, and each one different for each *j*. This means we can demand that such *n* coefficients fulfill the following:

(A6) $$\begin{array}{ccc} |\psi \rangle & = & \frac{1}{\sqrt{n}}\sum_{u,v=1}^{n}|B_{uv}\rangle_{Ca}⊗\sum_{j=1}^{n}\sqrt{p_{j}}T_{jk(j),uv}|\phi_{j}\rangle_{\left\{2i\right\}}|\psi_{j}\rangle_{2i}{|k\left(j\right)\rangle }_{b} \end{array}$$

where k(j) is an one-to-one function on the set {1,2,…,n}. For $n=2^{q},q\in Z$, such a selection is easily possible. Because only *n* coefficients appear when the terms $\sqrt{p_{j}}|\phi_{j}\rangle_{\left\{2i\right\}}|\psi_{j}\rangle_{2i}{|j\rangle }_{b}$ in (A6) are combined, and we can visualize such combination based on the following arrangement (where blocks of size *n* have been remarked here):

(A7)

There, each row states for each |Buv〉 which combinations for j,k appear in the second sum of (A6). Such coefficient arrangement could be constructed as follows. For the first block of rows for u=1, *n* rows were constructed for each *v*. Each one contains just *n* coefficients $\frac{1}{\sqrt{n}}$ allocated in 11,22,…,nn rows for v=1. In the other n−1 rows, entries different from zero just were moved cyclically one place to the right consecutively.

For the next n−1 blocks for each *u*, initially the last procedure is repeated. Nevertheless, we need to demand $|B_{uv}\rangle$ that will be orthonormal among them. Each column in the block j=1 is orthonormal; however, columns for j=2,…,n require modifications. In fact, by selecting the last $\frac{n}{2}$ different block-rows for the second block-column changing the signs to their entries, we obtain orthogonal columns among them and among those columns in the first block-column. By repeating the last procedure with the third block-column, changing the sign in the last second half-section with the same signs, we will obtain orthonormal vectors among them and with the two previous block-columns. Such procedure can be repeated with the remaining block-columns only if $n=2^{q},q\in Z$. Such block structure is affordable based on the last single method for $n=2^{q}$, not requiring the knowledge of the other involved states of Bob. If such $|B_{uv}\rangle_{Ca}$ basis selection is possible, then additionally there exists a set of local exchange ($\sum_{j,k(j)\neq i=1}^{n}{|i\rangle }_{b}\langle i|+{|k\left(j\right)\rangle }_{b}\langle j|+{|j\rangle }_{b}\langle k\left(j\right)|$) and phase transformations ($\sum_{j\neq i=1}^{n}{|i\rangle }_{b}\langle i|-{|j\rangle }_{b}\langle j|$), $L_{uv}^{b}$ on *b*, fulfilling:

(A8) $$\begin{array}{ccc} |\psi \rangle & = & \frac{1}{n}\sum_{u,v=1}^{n}|B_{uv}\rangle_{Ca}⊗L_{uv}^{b}\sum_{j=1}^{n}\sqrt{p_{j}}|\phi_{j}\rangle_{\left\{2i\right\}}|\psi_{j}\rangle_{2i}{|j\rangle }_{b} \end{array}$$

then, Alice applies the semi-local operation $T^{-1}=\left(T_{u^{\prime }v^{\prime },uv}^{-1}\right)$ on her qubits *C* and *a*, obtaining:

(A9) $$\begin{array}{ccc} |\psi^{\left(1\right)}\rangle & = & \frac{1}{n}\sum_{u,v=1}^{n}{|uv\rangle }_{Ca}⊗L_{uv}^{b}\sum_{j=1}^{n}\sqrt{p_{j}}|\phi_{j}\rangle_{\left\{2i\right\}}|\psi_{j}\rangle_{2i}{|j\rangle }_{b} \end{array}$$

The following development can be achieved using delayed measurements [76] or controlled operations. They commonly require interactions between faraway resources, which implies some of them can be moved from their locations using additional classical communication operations. Instead, we will use projective measurements and corrections. Thus, Alice measures their qubits C,a obtaining the outcomes ${|u\rangle }_{C}$ and ${|v\rangle }_{a}$, respectively. Using classical communication, Alice applies the controlled operation $C^{C}{L^{b}}^{†}\equiv \sum_{u,v=1}^{n}{|u\rangle }_{C}\langle u|⊗{|v\rangle }_{a}\langle v|⊗{L_{uv}^{b}}^{†}$ or otherwise better as is required by the locality, she measures the state ${|uv\rangle }_{Ca}$ and shares those outcomes with Bob${}_{i}$ who applies ${L_{uv}^{b}}^{†}$ locally. The outcome is as follows:

(A10) $$\begin{array}{ccc} |\psi^{\left(2\right)}\rangle & = & {|uv\rangle }_{Ca}⊗\sum_{j=1}^{n}\sqrt{p_{j}}|\phi_{j}\rangle_{\left\{2i\right\}}|\psi_{j}\rangle_{2i}{|j\rangle }_{b} \end{array}$$

thus, Bob${}_{i}$ applies the controlled operation $C_{i}^{b}U_{i_{2i}}$:

(A11) $$\begin{array}{ccc} |\psi^{\left(3\right)}\rangle & = & {|uv\rangle }_{Ca}⊗(\sum_{i\neq j=1}^{n}\sqrt{p_{j}}|\phi_{j}\rangle_{\left\{2i\right\}}|\psi_{j}\rangle_{2i}{|j\rangle }_{b}+\sqrt{p_{i}}|\phi_{i}\rangle_{\left\{2i\right\}}U_{i_{2i}}|\psi_{i}\rangle_{2i}{|i\rangle }_{b}) \end{array}$$

Finally, qubit *b* is sent to Alice to perform the $SWAP_{Cb}$ operation:

(A12) $$\begin{array}{ccc} |\psi^{\left(4\right)}\rangle & = & {|vu\rangle }_{ab}⊗(\sum_{i\neq j=1}^{n}\sqrt{p_{j}}{|j\rangle }_{C}|\phi_{j}\rangle_{\left\{2i\right\}}|\psi_{j}\rangle_{2i}+\sqrt{p_{i}}{|i\rangle }_{C}|\phi_{i}\rangle_{\left\{2i\right\}}U_{i_{2i}}{|\psi_{i}\rangle }_{2i}) \end{array}$$

which, disregarding the qubits *a* and *b*, is the same state obtained by $C_{i}^{C}U_{i_{2i}}$.

## Appendix B. SWAP Operations between Faraway Non-Local Parties

We will show how to perform the $C_{SWAP_{2j,2k}}$ operation between the faraway parties 2j,2k (assuming they cannot be moved close together). We will use the entangled resource $|\beta_{00}\rangle_{ab}$, where qubit *a* is in possession of Bob${}_{j}$ and *b* is sent to Bob${}_{k}$:

(A13) $$\begin{array}{ccc} |\psi \rangle & = & \left(\sum_{i=1}^{n}\sqrt{p_{i}}{|i\rangle }_{C}{|0\rangle }_{\mathrm{even}}^{⊗n\backslash \{2i\}}{|\varphi_{0}^{i}\rangle }_{2i}\right)⊗{|\beta_{00}\rangle }_{ab} \end{array}$$

By rearranging the qubits 2j and *a* in the *j*-th term of (A13), and expressing it in terms of Bell stats basis:

(A14) $$\begin{array}{ccc} |\psi^{\left(1,j\right)}\rangle & = & \frac{\sqrt{p_{j}}}{2}{|j\rangle }_{C}{|0\rangle }_{\mathrm{even}}^{⊗n\backslash \{2j\}}\sum_{x,y\in \{0,1\}}|\beta_{xy}\rangle_{2j,a}Z_{b}^{x}X_{b}^{y}{|\varphi_{0}^{j}\rangle }_{b}\\ & & \;+\left(\sum_{j\neq i=1}^{n}\sqrt{p_{i}}{|i\rangle }_{C}{|0\rangle }_{\mathrm{even}}^{⊗n\backslash \{2i\}}{|\varphi_{0}^{i}\rangle }_{2i}\right)⊗{|\beta_{00}\rangle }_{ab} \end{array}$$

then, Alice and Bob${}_{0}$ apply the controlled operation $C_{j}^{C}\left(H_{2j}C^{2j}NOT_{a}\right)$ on their qubits (where, $C_{j}^{a}G_{b}\equiv {|j\rangle }_{a}\langle j|⊗G_{b}+\sum_{j\neq i=1}^{n}{|j\rangle }_{a}\langle j|⊗1_{b}$. Accordingly, we obtain:

(A15) $$\begin{array}{ccc} |\psi^{\left(2,j\right)}\rangle & = & \frac{\sqrt{p_{j}}}{2}{|j\rangle }_{C}{|0\rangle }_{\mathrm{even}}^{⊗n\backslash \{2j\}}\sum_{x,y\in \{0,1\}}{|xy\rangle }_{2j,a}Z_{b}^{x}X_{b}^{y}{|\varphi_{0}^{j}\rangle }_{b}\\ & & \;+\left(\sum_{j\neq i=1}^{n}\sqrt{p_{i}}{|i\rangle }_{C}{|0\rangle }_{\mathrm{even}}^{⊗n\backslash \{2i\}}{|\varphi_{0}^{i}\rangle }_{2i}\right)⊗{|\beta_{00}\rangle }_{ab} \end{array}$$

after, Bob${}_{2j}$ measures qubits 2j and *a* if the control register is ${|j\rangle }_{C}$, obtaining ${|x\rangle }_{2j}$ and ${|y\rangle }_{a}$ using controlled quantum measurements [77,78]. Thus:

(A16) $$\begin{array}{ccc} |\psi^{\left(3,j\right)}\rangle & = & \sqrt{p_{j}}{|j\rangle }_{C}{|0\rangle }_{\mathrm{even}}^{⊗n\backslash \{2j\}}{|xy\rangle }_{2j,a}Z_{b}^{x}X_{b}^{y}{|\varphi_{0}^{j}\rangle }_{b}\\ & & \;+\left(\sum_{j\neq i=1}^{n}\sqrt{p_{i}}{|i\rangle }_{C}{|0\rangle }_{\mathrm{even}}^{⊗n\backslash \{2i\}}{|\varphi_{0}^{i}\rangle }_{2i}\right)⊗{|\beta_{00}\rangle }_{ab} \end{array}$$

Using controlled classical communication, the measurement outcomes are shared with Bob${}_{2k}$ if the control register is ${|j\rangle }_{C}$ to perform the operation $X_{b}^{y}Z_{b}^{x}$ and, subsequently, $SWAP_{2k,b}$, all on his qubits. Similarly, Bob${}_{2j}$ applies $C_{j}^{C}X_{2j}^{x}$ and $C_{j}^{C}X_{a}^{y}$. It gives:

(A17) $$\begin{array}{ccc} |\psi^{\left(4,j\right)}\rangle & = & \sqrt{p_{j}}{|j\rangle }_{C}{|0\rangle }_{\mathrm{even}}^{⊗n\backslash \{2k\}}{|00\rangle }_{ab}{|\varphi_{0}^{j}\rangle }_{2k}\\ & & \;+\left(\sum_{j\neq i=1}^{n}\sqrt{p_{i}}{|i\rangle }_{C}{|0\rangle }_{\mathrm{even}}^{⊗n\backslash \{2i\}}{|\varphi_{0}^{i}\rangle }_{2i}\right)⊗{|\beta_{00}\rangle }_{ab} \end{array}$$

Finally, Bob${}_{2j}$ uses controlled quantum measurements again, when the control register is different from ${|j\rangle }_{C}$ to measure the qubit *a*, obtaining ${|z\rangle }_{a}$ as outcome. He performs $C^{C}X_{a}^{z}$ and uses controlled classical communication to share the outcome to Bob${}_{2k}$ who performs $X_{b}^{z}$. It provides the following state:

(A18) $$\begin{array}{c} |\psi^{\left(5,j\right)}\rangle =\left(\sqrt{p_{j}}{|j\rangle }_{C}{|0\rangle }_{\mathrm{even}}^{⊗n\backslash \{2k\}}|\varphi_{0}^{j}\rangle_{2k}+\sum_{j\neq i=1}^{n}\sqrt{p_{i}}{|i\rangle }_{C}{|0\rangle }_{\mathrm{even}}^{⊗n\backslash \{2i\}}{|\varphi_{0}^{i}\rangle }_{2i}\right)⊗{|00\rangle }_{ab} \end{array}$$

then, by disregarding ${|00\rangle }_{ab}$ we obtain such a process to repeat it now with each one of the other parties different from 2j and 2k. Finally, we obtain:

(A19) $$\begin{array}{ccc} |\Psi_{k}{\rangle ⊗|0\rangle }_{\mathrm{even}}^{⊗n\backslash \{2k\}} & = & \left(\sum_{i=1}^{n}\sqrt{p_{i}}{|i\rangle }_{C}⊗{|\varphi_{0}^{i}\rangle }_{2k}\right)⊗{|0\rangle }_{\mathrm{even}}^{⊗n\backslash \{2k\}} \end{array}$$

which fits with $|\Psi_{k}\rangle$ in (16).

## Appendix C. Averages and Variances of *P** (1 − *P_C_*) Probability for the Pattern Matching Procedure

If $\left\{\theta_{i},\phi_{i}|i\in K\right\}$ are uniformly distributed on the Bloch sphere, by using the Haar measure on it [54], the following expected values can be obtained from a direct calculation (the line over indicates the expected value function):

(A20) $$\begin{array}{ccc} \bar{\langle \varphi^{*}|\varphi_{0}^{i}\rangle }\; & = & \frac{1}{8\pi }\int_{0}^{2\pi }\int_{0}^{2\pi }\left|\sin\theta_{i}\right|\langle \varphi^{*}|\varphi_{0}^{i}\rangle d\theta_{i}\phi_{i}=0 \end{array}$$

(A21) $$\begin{array}{ccc} \bar{|\langle \varphi^{*}|\varphi_{0}^{i}\rangle {|}^{2}} & = & \frac{1}{8\pi }\int_{0}^{2\pi }\int_{0}^{2\pi }|\sin\theta_{i}\left|\right|\langle \varphi^{*}|\varphi_{0}^{i}\rangle {|}^{2}d\theta_{i}\phi_{i}=\frac{1}{2} \end{array}$$

(A22) $$\begin{array}{ccc} \bar{{\langle \varphi^{*}|\varphi_{0}^{i}\rangle }^{2}}\; & = & \frac{1}{8\pi }\int_{0}^{2\pi }\int_{0}^{2\pi }\left|\sin\theta_{i}\right|{\langle \varphi^{*}|\varphi_{0}^{i}\rangle }^{2}d\theta_{i}\phi_{i}=\frac{1}{4}\left(1+\cos\theta^{*}\right) \end{array}$$

(A23) $$\begin{array}{ccc} \bar{|\langle \varphi^{*}|\varphi_{0}^{i}\rangle {|}^{4}} & = & \frac{1}{8\pi }\int_{0}^{2\pi }\int_{0}^{2\pi }|\sin\theta_{i}\left|\right|\langle \varphi^{*}|\varphi_{0}^{i}\rangle {|}^{4}d\theta_{i}\phi_{i}=\frac{1}{3} \end{array}$$

there, Bloch sphere is integrated twice to consider all mathematical possible two levels quantum states. Accordingly, an important issue in this development is as follows:

(A24) $$\begin{array}{c} \bar{|\sum_{i\in K}\langle \varphi^{*}|\varphi_{0}^{i}\rangle {|}^{2}}=\bar{\sum_{i\in K}{\left|\langle \varphi^{*}|\varphi_{0}^{i}\rangle \right|}^{2}} \end{array}$$

Then, due *P* could be expressed as:

(A25) $$\begin{array}{ccc} P & = & P^{*}\left(1-P_{C}\right)=\frac{1}{b}\sum_{i\in K}|\langle \varphi^{*}|\varphi_{0}^{i}\rangle {|}^{2}-\frac{1}{b^{2}}{\left|\sum_{i\in K}\langle \varphi^{*}|\varphi_{0}^{i}\rangle \right|}^{2} \end{array}$$

by considering (A20)–(A23), it is easy to show that:

(A26) $$\begin{array}{c} \mu_{P}=\bar{P^{*}\left(1-P_{C}\right)}=\left\{\begin{array}{cc} \frac{b-1}{2b}, & |\varphi^{*}\rangle \notin {\{|\varphi_{0}^{i}\rangle }_{2k}\left|i\in K\right\}\\ \frac{b^{2}-1}{2b^{2}}, & |\varphi^{*}\rangle \in {\{|\varphi_{0}^{i}\rangle }_{2k}\left|i\in K\right\} \end{array}\right. \end{array}$$

The first expression is obtained by considering the expected value of the expression and subsequently changing each term by the corresponding outcomes in (A20)–(A23). Instead, the second one, when one term *k* matches with $|\varphi^{*}\rangle$, 1 is substituted: $\langle \varphi^{*}|\varphi_{0}^{k}\rangle =1$. Similarly, continuing the calculation:

(A27) $$\begin{array}{ccc} \sigma_{P}^{2} & = & \bar{{\left(P^{*}\left(1-P_{C}\right)\right)}^{2}}-{\bar{P^{*}\left(1-P_{C}\right)}}^{2}\\ & = & \left\{\begin{array}{cc} \frac{\left(b-1\right)\left(4b+3\cos\left(\theta^{*}\right)\left(\cos\left(\theta^{*}\right)+2\right)-1\right)}{48b^{3}}, & |\varphi^{*}\rangle \notin {\{|\varphi_{0}^{i}\rangle }_{2k}\left|i\in K\right\}\\ \frac{\left(b-1\right)\left(3\left(b-2\right)\cos^{2}\left(\theta^{*}\right)+6\left(b+2\right)\cos\left(\theta^{*}\right)+b\left(4b-5\right)+22\right)}{48b^{4}}, & |\varphi^{*}\rangle \in {\{|\varphi_{0}^{i}\rangle }_{2k}\left|i\in K\right\} \end{array}\right. \end{array}$$
